# A ddRAD-based genetic map and its integration with the genome assembly of Japanese eel (*Anguilla japonica*) provides insights into genome evolution after the teleost-specific genome duplication

**DOI:** 10.1186/1471-2164-15-233

**Published:** 2014-03-26

**Authors:** Wataru Kai, Kazuharu Nomura, Atushi Fujiwara, Yoji Nakamura, Motoshige Yasuike, Nobuhiko Ojima, Tetsuji Masaoka, Akiyuki Ozaki, Yukinori Kazeto, Koichiro Gen, Jiro Nagao, Hideki Tanaka, Takanori Kobayashi, Mitsuru Ototake

**Affiliations:** 1 National Research Institute of Fisheries Science, Fisheries Research Agency, Yokohama-shi, Kanagawa 236-8648, Japan; 2 National Research Institute of Aquaculture, Fisheries Research Agency, Minami-ise-cho, Mie 516-0193, Japan; 3 Fisheries Research Agency, Yokohama-shi, Kanagawa 220-6115, Japan; 4 Present address: Seikai National Fisheries Research Institute, Fisheries Research Agency, Nagasaki-shi, Nagasaki 851-2213, Japan

**Keywords:** Japanese eel, Genetic map, ddRAD-seq, Ion PGM, Synteny, Teleost-specific whole genome duplication

## Abstract

**Background:**

Recent advancements in next-generation sequencing technology have enabled cost-effective sequencing of whole or partial genomes, permitting the discovery and characterization of molecular polymorphisms. Double-digest restriction-site associated DNA sequencing (ddRAD-seq) is a powerful and inexpensive approach to developing numerous single nucleotide polymorphism (SNP) markers and constructing a high-density genetic map. To enrich genomic resources for Japanese eel (*Anguilla japonica*), we constructed a ddRAD-based genetic map using an Ion Torrent Personal Genome Machine and anchored scaffolds of the current genome assembly to 19 linkage groups of the Japanese eel. Furthermore, we compared the Japanese eel genome with genomes of model fishes to infer the history of genome evolution after the teleost-specific genome duplication.

**Results:**

We generated the ddRAD-based linkage map of the Japanese eel, where the maps for female and male spanned 1748.8 cM and 1294.5 cM, respectively, and were arranged into 19 linkage groups. A total of 2,672 SNP markers and 115 Simple Sequence Repeat markers provide anchor points to 1,252 scaffolds covering 151 Mb (13%) of the current genome assembly of the Japanese eel. Comparisons among the Japanese eel, medaka, zebrafish and spotted gar genomes showed highly conserved synteny among teleosts and revealed part of the eight major chromosomal rearrangement events that occurred soon after the teleost-specific genome duplication.

**Conclusions:**

The ddRAD-seq approach combined with the Ion Torrent Personal Genome Machine sequencing allowed us to conduct efficient and flexible SNP genotyping. The integration of the genetic map and the assembled sequence provides a valuable resource for fine mapping and positional cloning of quantitative trait loci associated with economically important traits and for investigating comparative genomics of the Japanese eel.

## Background

Next-generation sequencing platforms permit for the cost-effective sequencing of whole or partial genomes in a relatively short time, which is greatly accelerating genomic research in non-model organisms. In particular, single nucleotide polymorphism (SNP) genotyping by restriction-site associated DNA sequencing (RAD-seq) is a powerful approach for developing a large number of genetic markers, for constructing a high-density genetic map and for studying the genetic architecture of complex traits [1-6]. This approach sequences short targets that immediately flank sites for a particular restriction enzyme throughout a genome; this produces a reduced representation of the genome, allowing oversequencing of the nucleotides next to the restriction sites and detection of SNPs. This approach can rapidly and cost-effectively discover SNPs compared with whole-genome sequencing (reviewed in [7]); nevertheless, a massive amount of sequence data is required to increase the read depth for more accurate SNP calling. Therefore, in previous studies, RAD-seq has generally been performed using Illumina sequencing platforms, such as the Genome Analyzer IIx (GAIIx) and HiSeq series [1-6]. Recently, double-digest RAD-seq (ddRAD-seq) has been developed as a simple and robust method [8]. Unlike the RAD-seq, this approach uses two restriction enzyme comprising a rare-cutting one and frequently-cutting one and avoids random shearing of the DNA. Such precise and repeatable size selection allows stable shared region recovery across samples (or individuals). Thus, ddRAD-seq produces more effective SNP genotyping compared with RAD-seq, even using a relatively small number of reads generated by a small next-generation benchtop sequencer, such as an Ion Torrent Personal Genome Machine (Life Technologies Corporation, Carlsbad, CA, USA), which was launched in early 2011 [9]. This platform performs semiconductor sequencing that relies on the detection of pH changes induced by the release of a proton upon the incorporation of a nucleotide during strand synthesis [10] and generates over 500 million reads per run at 4.5 hours. The Ion PGM is a relatively new technology; therefore, its output, including quality and downstream application, has not yet fully matured [10]. However, as the sequencing cost (price per base) continues to drop, the platform will soon be applicable for small and medium-size laboratories [11].

The Japanese eel (*Anguilla japonica*) is one of the most widely cultivated fish species in East Asian countries; however, the farming of this species has been completely dependent on seeds from nature, and broodstock domestication on a commercial scale has yet to be fully developed. Aiming for stable production of this species, the first techniques for artificial breeding were established in 1970s [12,13]. Thereafter, research has improved these techniques [14], and recently we succeeded in obtaining an F_2_ generation of this species (unpublished results). Although further improvements are required for large-scale commercial production, the broodstock domestication and selective breeding, including a DNA marker-assisted selection based on quantitative trait loci (QTLs), is now within reach. A key step in such a genetic improvement scheme is an accumulation of genomic resources such as a draft genome sequence, transcriptome sequences and a genetic map. Recently, the draft genome of the Japanese eel was sequenced using Illumina platforms, yielding an assembly comprising 323,776 scaffolds and covering 1.15 Gb of the genome; gene prediction and annotation for this assembly are ongoing [15]. In addition, we constructed the first genetic linkage map of this species [16]. Integration of a genetic map and assembled sequences enable us to identify a chromosomal order of scaffolds in a draft assembly and provides a valuable resource for fine mapping and positional cloning of QTLs associated with economically important traits [17,18]. However, in the Japanese eel, because of the low resolution of the genetic map, the chromosomal order of scaffolds in the current assembly is unknown, which has limited the use of the genomic sequences.

In addition to assisting in genetic studies, a genome map integrating a genetic map and assembled sequences is also a powerful tool for investigating comparative genomics [19,20]. Eel species are one of the most distantly related groups to other teleost species and has diverged from the other teleosts in the basal lineage of the teleosts [21,22]. Therefore, comparing the genome of the Japanese eel with those of other fishes would provide an opportunity to understand the genomic architecture of ancestral teleosts and chromosomal rearrangement events that occurred during teleost genome evolution. Teleost genomes have experienced a teleost-specific whole genome duplication event (TGD) [23,24], which is thought to have triggered an acceleration of chromosomal rearrangements [4,25,26]. Indeed, Kasahara et al. [19] inferred that eight major chromosomal rearrangements occurred in the ancestral lineage of zebrafish (*Danio rerio*) and medaka (*Oryzias latipes*) after the TGD. In addition, the genome of the spotted gar (*Lepisosteus oculatus*), whose lineage diverged from the teleost lineage before the TGD, showed that the extent of conserved synteny between the gar and human (*Homo sapiens*) was higher than that between the gar and zebrafish, and the gar and the stickleback (*Gasterosteus aculeatus*), suggesting that the TGD accelerated the loss of ancestral syntenies [4]. Given the phylogenic position among these fishes, the TGD and subsequent chromosomal rearrangements are presumed to have occurred over a relatively short period [19]. The eel lineage diverged from other teleost lineage within this period [21,22]. Therefore, comparative genomics between the Japanese eel and model fishes provides new insights into the genome evolution soon after the TGD.

Here, we generated a ddRAD-based genetic map of the Japanese eel using an Ion PGM sequencer. The map comprised 19 linkage groups with a total of 2,672 SNP markers and 115 Simple Sequence Repeat (SSR) markers and was integrated with the current genome assembly to identify the chromosomal order of the scaffolds. In the current assembly, 151 Mb of the scaffolds has been anchored on the 19 linkage groups, and 70 Mb of them have been ordered on the linkage groups. Comparisons of the genomes between the Japanese eel and model fishes showed a highly conserved synteny among teleosts and revealed the history of genome evolution in the basal lineage of the teleosts.

## Methods

### Mapping population

Forty individuals of a wild-caught glass eel (Average of total length = 56.9 mm, body weight = 185.3 mg) of the Japanese eel were purchased from commercial fisheries in Japan. The sex ratio of this species is skewed towards the male under ordinary culture conditions [27,28]. Therefore, half of them were feminized by feeding a diet containing estradiol-17β for 5 months [29], and cultivated fishes were used as a dam; the remaining male fish were not treated. These female and male eels were cultivated in freshwater for 2–3 years and then acclimated to seawater. Hormonal treatment was carried out for artificial maturation, and a mapping population was obtained by *in vitro* crossing, as described previously [16,30,31]. Ninety-two full-sib progeny from the single cross were used to develop a linkage map. This project was conducted in accordance with the Guidelines for Animal Experimentation of the National Research Institute of Aquaculture (NRIA), and all animal procedures were approved by the Institutional Animal Care and Use Committee of the NRIA.

### ddRAD library preparation and sequencing

Muscle tissues of the parents were preserved in TNES urea buffer (10 mM Tris–HCl, pH 7.5, 125 mM NaCl, 10 mM EDTA, 1% sodium dodecyl sulfate, 8 M urea) at room temperature until DNA preparation. Genomic DNA was extracted from the stored samples using the standard phenol-chloroform technique. Genomic DNA of 198–457 days post-hatch juveniles (glass eels) was isolated from muscle tissues using the QuickGene-800 extraction platform (Fujifilm, Tokyo, Japan) with a QuickGene DNA tissue kit S (DT-S). Our ddRAD libraries were constructed using a method of combining a normal ddRAD-seq protocol [8] with an amplicon tagging protocol for Access Array technology (Fluidigm, South San Francisco, CA, USA) (also see Additional file 1: Figure S1). Briefly, 500 ng of DNA template from each individual was double-digested with BamHI-HF (10 units per reaction, New England Biolabs Inc., Beverly, MA, USA) and MspI (10 units per reaction, New England Biolabs Inc.) at 37°C for 1 h. We found approximately 100,000 cut sites of BamHI-HF (G|GATCC) and ~1,500,000 of MspI (C|CGG) in the current genome assembly of the Japanese eel [15]. Each fragmented sample was purified using the MinElute PCR purification kit (Qiagen GmbH, Hilden, Germany), eluted with 30 μL of TE buffer (pH 8.0). Fragments were size-selected using the Pippin Prep (2% agarose cartridge, Sage Science, Beverly, MA, USA) under a “broad” setting with a mean of 150 bp and range of 120–180 bp. After size-selection, each sample was purified using Agencourt AMPure XP beads (Beckman Coulter Inc., Brea, CA, USA). Purified samples were then ligated to CS1-tagged and CS2-tagged adapters, consisting of the common sequence tag 1 (CS1) and the common sequence tag 2 (CS2) of Access Array, respectively. The CS1-tagged adapter binds to overhangs generated by BamHI, and the CS2-tagged adapter contains overhangs compatible with an MspI site. Adapter ligation was performed in a 40-μL reaction volume containing 25 μL of DNA samples, 2 μL of CS1-tagged adapter (100 nM), 4 μL of CS2-tagged adapter (100 nM), 1 μL of T4 ligase (1 U/μL, Invitrogen, Carlsbad, CA, USA) and 8 μL of 5 × T4 ligation buffer (Invitrogen). The reaction was incubated at room temperature for 1 h, and heat-inactivate The T4 ligase was then heat inactivated at 65°C for 30 min. After adapter ligation, each DNA samples was purified using Agencourt AMPure XP beads and amplified using KAPA HiFi polymerase (KAPA biosystems, Woburn, MA, USA) with 400 nM primers of Access Array Barcode Library for Ion Torrent PGM Sequencer-96 (Fluidigm). The following PCR protocol was used: initial denaturation at 95°C for 5 min; eight cycles of 95°C for 15 sec, 60°C for 30 sec and 72°C for 1 min; followed by a final extension period at 72°C for 3 min. The amplified libraries were purified using Agencourt AMPure XP beads and quantified using the Qubit fluorometer with a Quant-it dsDNA HS kit (Invitrogen). The libraries of the parents and the 92 progeny were pooled (two individuals per library for the parents and 6–16 individuals per library for the progeny). The quality, size and concentration of the pooled libraries were finally determined using the 2100 Bioanalyzer with a high sensitivity DNA chip (Agilent technologies, Waldbronn, Germany), and the library was diluted before template preparation. Emulsion PCR and Ion Sphere Particles enrichment were carried out using the Ion OneTouch system with an Ion OneTouch 200 Template kit v2 DL (Life Technologies Corporation). The 200 template samples were sequenced on 318 chips using the PGM platform with an Ion PGM 200 sequencing kit (Life Technologies Corporation). Data from the PGM runs were processed using the Ion Torrent Suite 3.4.2 software to generate sequenced reads.

### Creation of the reference sequences and read mapping

Raw reads were filtered to discard those of low quality and sorted to individuals according to the barcode sequences of the Access Array using the process_radtags program in Stacks [32]. The processes were conducted using the following options: restriction enzyme of BamHI, rescue barcodes and RAD-tags, discard reads with low quality scores, remove any read with an uncalled base and truncate final read length of 100 bp. To generate reference sequences for SNP discovery, the processed ddRAD-tags derived from the parents were clustered using USEARCH [33] with an identity threshold of 0.75. Centroid sequences derived from the clusters (the cluster size was ≥50, were used for read mapping as reference sequences. The processed reads derived from both of the parents and the 92 progeny were mapped to the reference sequence using CLC Genomics Workbench (CLC Bio, Aarhus, Denmark) with the following parameters: mismatch cost of 2, insertion cost of 3, deletion cost of 3, length fraction of 0.95, similarity of 0.95 and non-specific match handling of ignore. The output BAM files of each individual were sorted and indexed using SAMtools [34].

### SNP calling and genotyping

SNPs were called using Unified Genotyper in the Genome Analysis Toolkit (GATK) [35], and the variants were filtered as follows: cluster window size of 10, quality of depth (QD) of <5 and genotype quality (GQ) of <30. For each given SNP site, the genotypes were labeled as “A” for a homozygous genotype of reference alleles, “B” for homozygous genotype of alternate non-reference alleles, and “H” for the heterozygous genotype. The low quality (QD < 5) and clustered SNP sites were removed from the genotype data, and the genotypes having lower reliability (GQ < 30) were replaced by a missing value.

### Genotyping for SSR markers

To clarify the correspondence between the previous linkage map [16] and the present map, we mapped SSR markers located in the previous map to the present map. In addition, we developed novel SSR markers using a Roche 454 GS FLX Titanium platform. We constructed a shotgun library derived from a single individual of the Japanese eel and performed 454-pyrosequencing according to the manufacturer’s protocol (Roche 454 Life Sciences, Branford, CT, USA). *De novo* assembly of the processed reads was performed using the Newbler assembler 2.6 with default parameters. Auto-primer [36] was used to identify SSRs and design primer from the resultant contigs. PCR reactions and genotyping were performed as previously described [16].

### Linkage map construction

To construct a linkage map of the Japanese eel, heterozygous markers in either one of the parents were used as a pseudo-testcross [37]. Linkage analyses were conducted for markers genotyped in at least 88 of 92 (>95%) individuals using the R/qtl package [38]. Markers showing significant Mendelian segregation distortion (*χ2* test, *P* < 0.001, d.f. = 1) were excluded. For all markers, pairwise recombination estimates and logarithm of odds (LOD) scores were calculated, and marker pairs were then assigned to linkage groups at a LOD threshold of 7.0. Local marker order was determined by multipoint analysis. Map distances (cM) were calculated using the Kosambi function. Following the initial mapping, potential genotyping errors that may be identified through apparent tight double-crossovers were calculated, and a doubtful genotype having an error LOD of >2 was replaced by a missing value. Subsequently, a linkage map was reconstructed using the error corrected dataset, including markers genotyped in at least 88 of 92 (>95%) individuals. The resultant map was drawn using the perl script of genetic_mapper revision 0.4 [39], with a modification of color images. The corrected length of the linkage map (*L*) was estimated by multiplying the length of each linkage group by (*m* + 1)/(*m* - 1), where *m* is the number of markers in the linkage group [40]. The genome coverage of the linkage map (*c*) was next estimated by calculating *c* = 1 - *e*^*-2dn/L*^, where *d* is the average interval of markers, *n* is the number of markers, and *L* is the length of the linkage map estimated above.

### Anchoring scaffolds in the Japanese eel genome assembly to the linkage map

To identify the chromosomal order of scaffolds in the current genome assembly of the Japanese eel, we anchored the scaffolds to the linkage map using RAD-tag sequences for SNP markers and contig sequences for SSR markers. The eel draft sequence was obtained from the eel genome website [41]. To find specific scaffolds associated with the sequences of SNP and SSR markers, these sequences were BLAST searched against the draft genome data with a cutoff *E-*value of *1e-20*. In cases where the query hit two or more scaffolds with a less than two-fold difference in the bit score, we did not assign any scaffold. The relative order between anchored scaffolds on each chromosome was determined by the following rule. The order of scaffolds was initially determined in accordance with the order of the anchor points to scaffolds on the female linkage map. If two or more scaffolds had the same anchor point, the largest scaffold was used for ordering. Some scaffolds had the same anchor point on female map while their anchor points on male map were genetically separated. In such cases, if the sum length of these scaffolds was longer than that of a scaffold whose length was the longest in the same anchor point, these scaffolds were ordered according to the male map. Otherwise the largest scaffold was ordered. In the present study, the scaffolds anchored only to the male map were not used for ordering, because the relative order between these scaffolds and that anchored only to the female map was indeterminate.

### Sequence comparison

To examine the conservation of syntenies between the Japanese eel and zebrafish, and the Japanese eel and medaka, we downloaded repeat-masked genomic sequences of zebrafish and medaka from the UCSC Genome browser [42]. We then excluded sequences that have not been mapped on chromosomes in each species from the dataset. For the Japanese eel, the sequences of scaffolds anchored to chromosomes (linkage groups) were used for sequence alignments. Before alignment, repetitive elements in the anchored scaffolds were masked using RepeatMasker [43]. The anchored scaffold sequences of individual chromosomes were firstly brought together into single-FASTA files with gap sequences of (N)_10,000_ between the scaffolds. This was to prevent a misdetection of a conserved sequence segment that lies astride two different scaffolds in the alignment processes. Then, the single-FASTA files of individual chromosomes were integrated into a multi-FASTA file. To detect triplets of conserved sequence segments among the three species, multiple genome alignments were performed using the progressiveMauve aligner [44], with the following parameters: seed weight of 20 and use a family of spaced seeds. Data for the conserved sequence segments in the output backbone files were used for constructing Oxford grids. To identify homologous regions in the spotted gar genome, the eel sequences associated with the conserved segments were searched against the spotted gar draft genome assembly using a BLAT program at the Pre Ensembl website [45], with default parameters. In cases where the query matched two or more chromosomal regions with a less than two-fold difference in the bit score, the match was considered insignificant.

## Results

### Double-digest RAD sequencing using Ion PGM platform

Ion PGM sequencing generated 6.7 million raw reads from the mapping parents (Additional file 2: Table S1). After trimming the CS1-tgged and CS2-tagged adapters and filtering the low quality reads, 2,287,121 and 2,310,898 ddRAD-tags were obtained from the dam and sire, respectively. These tags from the parents were clustered into 9,634 RAD loci, of which the centroid sequences were used as reference sequences for SNP discovery. The reference sequences of the RAD loci are listed in Additional file 3. Then, Ion PGM sequencing yielded 96.3 million raw reads from the 92 progeny (Additional file 2: Table S1). After read processing, approximately 780,000 (SD: 194,262) tags were obtained per individual. All ddRAD-tags obtained from the parents and the progeny were submitted to the CLC Genomics Workbench and mapped to the reference sequences, resulting in 1,891,691 tags being mapped in the dam (on average, 195.6× coverage per locus), 1,943,489 tags in the sire (201.1×), and approximately 600,000 (SD: 145,465) tags in each progeny (62.5×, SD: 15.0). The raw reads of the ddRAD-seq have been deposited in the DDBJ Sequence Read Archive (DRA) under accession number [DDBJ:DRA001739].

### SNP detection and genotyping

SNP detection and genotyping were performed using the Unified Genotyper in the GATK with default options, resulting in the identification of 14,397 biallelic SNP sites in the 9,634 loci. According to QD annotation, the SNP sites having low probability were excluded from the dataset. Additionally, the clustered SNP sites, which are often generated by misalignment in repetitive or homopolymeric regions, were also excluded from the dataset. As a result, 10,943 SNP sites passed through the filtration and were used as putative markers for constructing the linkage map. Among them, 2,694 markers were heterozygous in either one of the parents and were genotyped for sufficient numbers of progeny. Of these markers, 23 markers were significantly distorted from a Mendelian segregation ratio (1:1 in the backcross model) and were excluded from the dataset. The remaining 2,671 markers were informative and used for constructing the linkage map. Details of SNP detection are shown in Additional file 2: Table S2.

### SSR genotyping

To clarify the correspondence between the previous linkage map [16] and the present map, we chose 106 SSR markers that were distributed genome-wide in the previous map. Additionally, to increase the number of SSR markers, a shotgun library was sequenced using a Roche/454 GS FLX Titanium platform, which generated 1,005,279 reads (average length = 254 bp) representing 255,755,881 bp. The raw sequencing data produced in this work have been deposited in the DRA under accession number [DDBJ:DRA001189]. After trimming the adapters and filtering the low quality and small reads, the high quality reads were assembled into 10,502 contigs (Average length of 374 bp, N_50_ length of 456 bp). After detecting SSRs from the contigs, we developed 144 SSR markers using previously described methods [16]. Taken together, a total of 250 SSR markers were used for genotyping. To obtain informative markers for the mapping family, we examined the segregation type of these SSR loci in the parents, which showed that 124 markers exhibited polymorphisms. These markers were scored in the 92 progeny and tested for segregation distortion. In total, 115 markers were informative for the mapping family and were used to generate the linkage map together with the SNP markers. Details of primer sequences and DDBJ accessions [DDBJ:AB500964, AB860330-AB860372] are shown in Additional file 2: Table S3.

### A second-generation linkage map of the Japanese eel

In a backcross model for a linkage analysis, a marker segregation type of “B/H” is used as a synonym of that of “A/H”. We therefore converted the genotype “B” to “A” in the genotype data before linkage analyses. The genotype data for female and male linkage maps are shown in Additional file 2: Table S4, Table S5.

**Figure 1:**
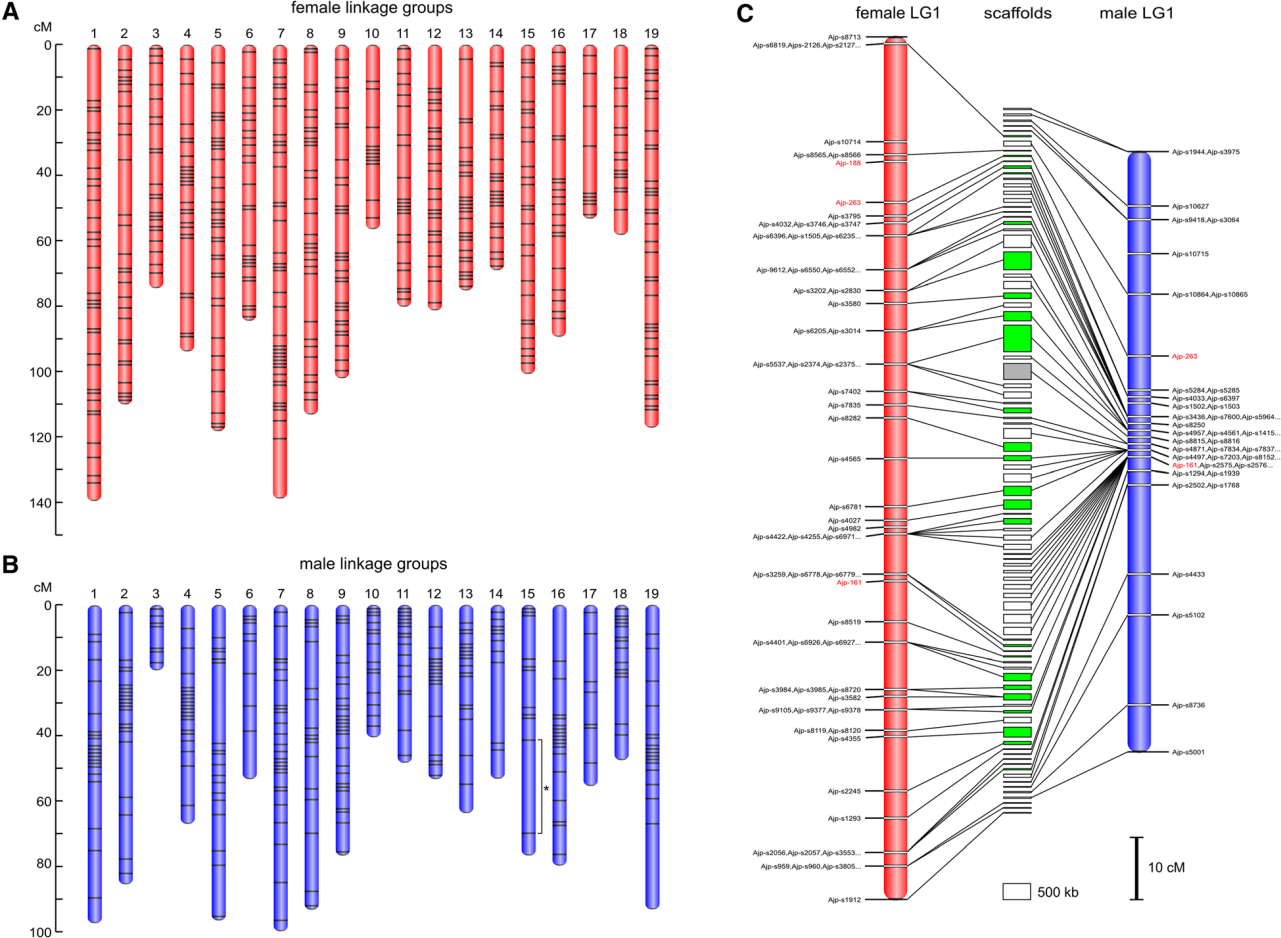
**Linkage maps of the Japanese eel and integration of the map and the assembled sequences. (A)** Female linkage map. Bars on each linkage group represent the loci of mapped SNP and SSR markers. Genetic distances (cM) were calculated using the Kosambi function. **(B)** Male linkage map. Detailed maps of female and male are shown in Additional file 1: Figure S2 and Figure S3. An asterisk shows that two marker loci are linked with a LOD threshold of 4.0. **(C)** Integration of the linkage map and the assembled sequences for LG1. Vertical red and blue bars represent female and male linkage group, respectively. SNP markers are black, and SSR markers are red. Boxes sandwiched between the female and male linkage groups represent scaffolds. Scaffolds for which the relative order on each chromosome has been determined are labeled in green. A scaffold that is mapped on other linkage groups is labeled in gray. This inconsistency could be caused by a misassembly in the current assembly. Transverse lines link the location of each marker on the genetic maps with its corresponding anchored scaffolds. Gaps between scaffolds are arbitrary because the actual distance between scaffolds is unknown. Scale bars represent 10 cM and 500 kb. Details of all chromosomes and anchored scaffolds are shown in Additional file 1: Figure S4.

The female map comprised 19 linkage groups with a total of 1,527 markers (1,428 SNP markers and 99 SSR markers) (Figure 1A, Table 1, Additional file 1: Figure S2, Additional file 2: Table S6). These 1,428 SNP markers were located in 1,004 RAD loci, accounting for 1.5 SNP sites per RAD sequence of 100 bp. The total length of the linkage groups was 1,748.8 cM, and the length of each linkage group ranged from 52.9 to 139.2 cM (mean 92.0 cM). The number of markers per linkage group varied from 32 to 108, with an average of 80 markers per linkage group, and the average distance spacing between two markers was 3.6 cM.

**Table 1:** Number of markers, genetic length and anchored scaffolds for each linkage group

**Linkage group**	**Total markers**	**Female map**		**Male map**		**Anchored scaffolds**	
		**Number of markers**	**Length (cM)**	**Number of markers**	**Length (cM)**	Number^*^	**Size (bp)**
1	154	83	139.2	73	96.9	86	10,779,947
2	185	109	109.7	84	85.1	80	11,549,278
3	127	60	74.1	70	19.6	46	6,166,433
4	193	100	93.5	102	66.7	84	11,926,296
5	144	88	117.9	58	96.2	73	8,932,484
6	133	72	84.1	63	53.0	52	6,456,950
7	227	106	138.4	127	99.5	100	14,079,542
8	163	89	112.8	79	92.9	77	7,915,729
9	204	101	101.7	108	76.3	95	11,854,887
10	123	63	56.1	66	40.2	59	7,250,929
11	152	86	79.8	75	48.0	64	5,754,071
12	154	95	80.9	65	53.0	60	8,740,852
13	131	67	74.8	67	63.4	57	5,716,409
14	133	71	68.7	63	52.9	61	8,348,650
15	88	71	100.4	22	76.3	46	4,849,938
16	151	87	89.0	69	79.4	67	6,927,157
17	54	32	52.9	22	55.1	21	915,430
18	123	68	57.9	60	47.2	54	3,975,438
19	148	79	116.9	73	92.8	69	9,131,346
Total	2,787	1,527	1748.8	1,346	1294.5	1,251	151,271,766

^*^Scaffolds that are anchored to two or more loci on the linkage map are included.

The male map initially consisted of 20 linkage groups including a total of 1,346 markers (1,243 SNP markers and 103 SSR markers) at the LOD threshold of 7.0 (data not shown). These 1,243 SNP markers were positioned in 933 RAD loci, accounting for 1.3 SNP site per RAD sequence. Of all the SNP markers on both maps, 574 markers in female and 555 markers in male were co-located within 396 common RAD loci. Using these SNP markers and SSR markers that were heterozygous in both parents, we identified homologous pairs of linkage groups of the female and male. Eighteen pairs of linkage groups exhibited one-to-one correspondence between the female and male maps, whereas two linkage groups in the male were homologous to linkage group 14 in the female. Moreover, markers located on the two linkage groups in the male were linked with a LOD threshold of 4.0. We therefore considered the two linkage groups as a single linkage group. Subsequently, the male map comprised 19 linkage groups (Figure 1B, Table 1, Additional file 1: Figure S3. Additional file 2: Table S6). This map spans 1,294.5 cM, and the size of each linkage group ranged from 19.6 to 99.5 cM (mean 68.1 cM). The number of markers per linkage group varied from 22 to 127, with an average of 71 markers per group, and the average distance between two markers was 4.1 cM.

Based on the homologous pairs of linkage groups of the female and male, the resultant map consisted of 19 linkage groups including a total of 2,787 markers (Table 1). In this map, all informative markers showed detectable linkage to at least one other marker. The total genetic distances of the female and male maps were 1,748.8 and 1,294.5 cM, respectively. Using the method of Chakravarti et al. [40], the corrected lengths of the maps were estimated at 1,890.0 cM and 1457.0 cM, which were converted into genome coverages of 85.4% and 84.8%, respectively.

### Integration of the genetic map and the assembled sequences

Targeting markers to specific regions of the scaffolds enabled us to identify the chromosomal order of the scaffolds in the Japanese eel genome assembly. Using BLAST searches, 2,260 of 2,672 SNP markers and 82 of 115 SSR markers sequences were assigned to 1,252 scaffolds covering 151 Mb (13% of the current assembly) (Figure 1C, Table 1, Additional file 1: Figure S4, Additional file 2: Table S6). Among them, 483 scaffolds covering 70 Mb (6%) were ordered to be consistent with the order of markers determined on the linkage maps. However, for most of the scaffolds, we were unable to identify their orientation on the linkage groups, because their sequence lengths of them were too short (average length was 145 kb), and genetically separated markers within the scaffold were rarely observed.

### Syntenic relationships between the Japanese eel and model fishes

**Figure 2:**
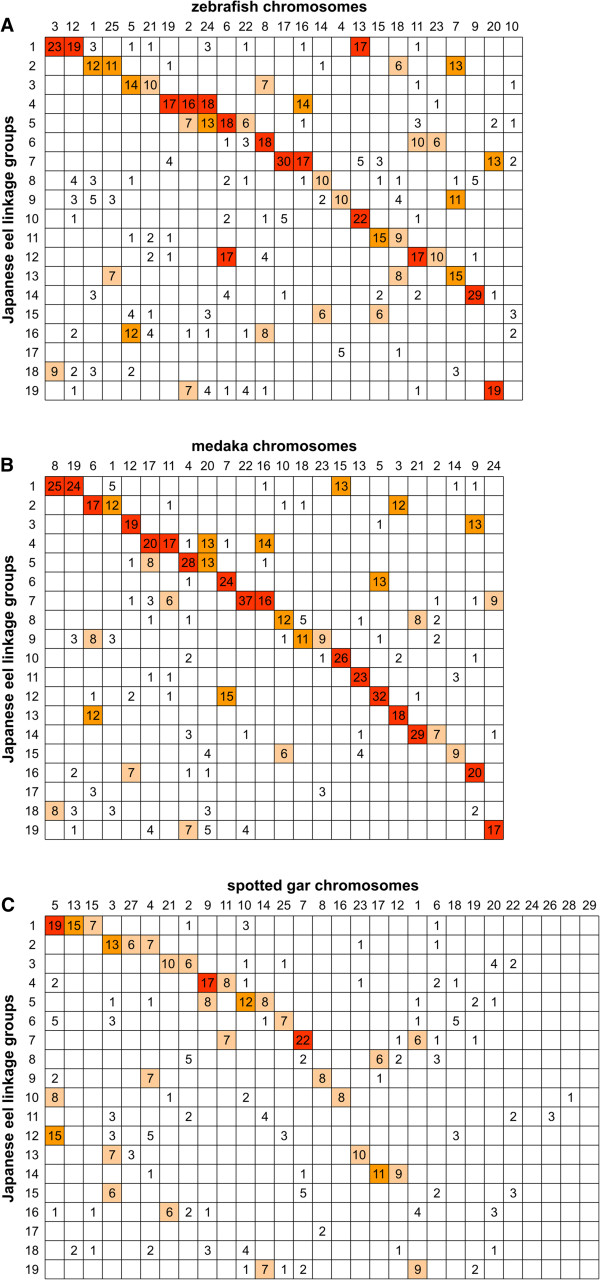
**Oxford grids showing conservation of syntenies between the Japanese eel and model fishes.** Conserved sequence segments are arrayed according to chromosome (or linkage group) for each species. Numbers in boxes indicate the number of the conserved sequence segments. **(A)** Comparison of the Japanese eel and zebrafish. **(B)** Comparison of the Japanese eel and medaka. **(C)** Comparison of the Japanese eel and the spotted gar. Details of the conserved sequence segments are listed in Additional file 2: Table S7.

Anchoring the genomic sequences to the chromosomes (linkage groups) of the Japanese eel made it possible to compare the conservation of synteny between the Japanese eel and other fishes. To compare the extent of conserved synteny among teleosts, we firstly identified 745 triplets of conserved sequence segments among the Japanese eel, zebrafish and medaka (Additional file 2: Table S7). These segments were arrayed in Oxford grids according to the chromosomes of each species (Figure 2). For comparison of the eel-zebrafish, the oxford grid showed that 582 of 745 (78%) segment pairs fell in 44 syntenic boxes, each comprising six or more segment pairs (Figure 2A). Moreover, 297 (40%) segment pairs were mapped into 15 syntenic boxes that had at least 16 segment pairs. Boxes with high number of segment pairs indicate a high degree of conserved synteny between the eel and zebrafish. In contrast, 163 (22%) segment pairs did not fall into syntenic boxes. This represents a trace of interchromosomal rearrangements after they diverged from their last common ancestor. For comparison of the eel-medaka, 637 of 745 (86%) conserved sequence segment pairs fell in a total of 41 syntenic blocks (Figure 2B). Among them, 392 (53%) segment pairs were contained in 17 highly syntenic boxes, while 108 (15%) segment pairs did not fall into syntenic boxes. These results suggest that the extent of conserved synteny between the eel and medaka is greater than that between the eel and zebrafish. As expected the degree of synteny between zebrafish and medaka was highly conserved compared with that between the eel and zebrafish, and the eel and medaka (Additional file 1: Figure S5).

We next analyzed the syntenic conservation between the genomes of the Japanese eel and the spotted gar, which is a model species that serves as an outgroup for exploring the mechanisms of evolution after the TGD [4]. The eel sequences derived from the 745 conserved sequence segments were searched against the gar draft genome assembly using the BLAT program, resulting in the 745 sequences of the eel being assigned to 453 unique sequences of the gar (Additional file 2: Table S7). Of these, 437 sequences have been mapped on the chromosomes of the gar. The oxford grid between the eel and gar showed that 295 of 437 (68%) putative orthologous sequence pairs fell in 31 syntenic boxes, each comprising of six or more ortholog pairs, while 142 (32%) ortholog pairs did not fall into syntenic boxes (Figure 2C). Although there were many traces of interchromosomal rearrangements between the two species, the analysis showed some one-to-two correspondences between these genomes. For example, most of the eel orthologs mapped on gar chromosome 21 (Loc21) were found on eel linkage group 3 (Aja3, 10 of 17 orthologs) and Aja16 (6 of 17 orthologs). In the Oxford grid between the eel and medaka, Aja3 and Aja16 had an orthologous relationship to medaka chromosome 12 (Ola12) and Ola9, respectively, which were previously shown to be paralogous derived by the TGD [19]. Thus, Aja3 and Aja16 are likely to be a paralogous chromosome pair generated by the TGD. One-to-two relationships between the eel and gar genomes were also observed in Loc4 and Aja2/Aja9, Loc9 and Aja4/Aja5, Loc11 and Aja4/Aja7, Loc14 and Aja5/Aja19, Loc17 and Aja8/Aja14, and Loc1 and Aja7/Aja19. These pairs of eel linkage groups (or fragmented regions of them) also may possibly be paralogous pairs generated by the TGD.

## Discussion

### Double-digest RAD sequencing using the next-generation benchtop sequencer

We combined the ddRAD-seq approach with Ion PGM sequencing to perform high-throughput SNP genotyping. To the best of our knowledge, this is the first report of constructing a highly dense genetic map using the Ion PGM platform. Compared with the RAD-seq approach, the ddRAD-seq permits greater robustness in region recovery [8]. Indeed, our simulation showed that 87-88% of 150,000,000 ddRAD-tags were sufficient to recover 12,190 regions at 117-124× in the ddRAD-seq approach (Additional file 2: Table S8). In contrast, 15-16% of 150,000,000 RAD-tags recovered 9,275 regions at 25-26× in the RAD-seq approach. Details of the comparison of the ddRAD-seq and RAD-seq approaches are shown in Additional file 4. Although the read throughput of the Ion PGM is lower than that of GAIIx and Hiseq sequencers, the cost-effective sequencing and the rapid runtime (4.5 hours per run) of this platform facilitates routine analysis. We therefore conducted ddRAD sequencing with a relatively small number of multiplexed samples (~16 samples were pooled in our study). This has the advantage of making the library at low cost, because it needs relatively fewer sequence primers (or adapters) attaching barcode sequences for individual samples. In fact, a maximum of 16 sequence primers were required for ddRAD sequencing for the parents and 92 progeny in our study (Additional file 2: Table S1). Moreover, our libraries were constructed using a unique method of combining the normal ddRAD-seq protocol with an amplicon tagging protocol that is widely used in amplicon sequencing assay (Additional file 1: Figure S1). According to the normal RAD-seq or ddRAD-seq protocols, the barcode sequences for multiplexed samples are directly attached to the sequence adapters. Therefore, if a reanalysis is conducted with a change in restriction enzyme pair to obtain other SNP makers, the sequence adapters need to be redesigned for individual samples. In contrast, our method required only a pair of adapters to be changed without redesigning the sequence primers for individual samples. Thus, this method permit flexible working.

### The second-generation genetic map of the Japanese eel and its integration with the assembled sequences

We constructed female and male genetic maps of the Japanese eel using double-digest RAD sequencing. The map has been improved considerably in the present study. While the previous genetic map contained 319 markers [16], the present map contains 2,672 SNP and 115 SSR markers. In addition, the present map represents a ~2.0-fold increase in map resolution (female: 3.6 cM, male: 4.1 cM) over the previous map (female: 7.2 cM, male: 6.3 cM). The correspondence between the linkage groups of the previous map and those of the present map are shown in Additional file 1: Figure S6. The previous map consisted of 22 linkage groups. In the present map, linkage group 20 (LG20) of the previous map was integrated with LG16. In addition, the linkage between the markers located on previous LG21 was disrupted, and the markers have been mapped on different linkage groups (LG3 and LG11 of the present map). The correspondences among previous LGs (LG13, LG17 and LG22) were unknown because of the absence of SSR markers. Consequently, the present map comprises 19 linkage groups, which corresponds with the karyotypes of the Japanese eel (2n = 38) [46,47].

The total map lengths were strikingly different between the sexes (the corrected map lengths were 1890.0 cM for female and 1457.0 cM for the male map). Such a sex difference in recombination has been described in various animals [48], although the molecular mechanisms of this phenomenon remain the subject of debate [49]. Among fishes, an overall lower recombination ratio has been identified in males of rainbow trout (*Oncorhynchus mykiss*) [50], zebrafish [51], and fugu (*Takifugu rubripes*) [20]. In particular, a strong suppression of recombination has been observed around the centromeres in these fishes. In the Japanese eel, the marker distribution on the male linkage map showed that the apparent recombination deserts were located near the center of the chromosome on 10 linkage groups: LG1, LG2, LG4, LG5, LG7, LG8, LG9, LG12, LG16 and LG19 (Additional file 1: Figure S7A). According to the tendency observed in other fishes, this result suggested that the Japanese eel has at least 10 meta- or submetacentric chromosomes. To confirm this, we performed a cytogenetic observation, as described previously [52]. The result showed that the karyotype of this species consisted of 10 meta- or submetacentric and 9 acrocentric chromosome pairs (Additional file 1: Figure S8). Thus, the recombination reduction around centromeres occurs during male meiosis of the Japanese eel, and it is likely that this phenomenon is widespread among teleosts. In the fugu, the recombination deserts around the centromeres were observed on not only male but also female; however, the suppressed regions on the female map were extremely narrow compared with that on the male map [20]. Although, in the Japanese eel, the recombination on the female map is more uniform than that of the male map, slight reductions of the recombination were observed on LG10 and LG18 (Additional file 1: Figure S7B). In addition, the recombination in these regions was also relatively suppressed on the male map (Additional file 1: Figure S7C). Thus, the markers within the recombination deserts in LG10 and LG18 of the female map may be located very close to the centromeres.

Using markers targeting specific scaffolds, 1,252 scaffolds in the current genome assembly of the Japanese eel were assigned to 19 linkage groups. The high-density genetic map, to which the genomic sequences are assigned, facilitates fine mapping and positional cloning of QTLs controlling important traits for eel aquaculture, such as growth rate, timing of metamorphosis and disease resistance (reviewed in [53]). The map will also contribute to the identification of candidate genes underlying these QTLs by analyzing synteny between this species and model fishes.

Furthermore, integration of the genetic map and the assembled sequences could improve the genome assembly. For instance, in the fugu genome project, 338 Mb (86%) of the fugu draft sequence were placed onto 22 chromosomes using 1,220 SSR markers, and the current assembly was generated by filling some gaps and by organizing scaffolds into chromosomes based on the genetic map [20]. In present study, 151 Mb (13%) of the eel assembled sequence was assigned to the linkage groups using 2,260 SNP markers and 82 SSR markers. The abundant markers were used for anchoring the scaffolds; however, the total number of anchored sequences was relatively small compared with that of fugu. This is because the current draft genome sequence of the Japanese eel is more fragmented; the N50 length is 53 kb in the Japanese eel assembly [15] compared with 858 kb in the fugu assembly version 4 (by our calculation). Additionally, we found that some markers assigned to the same scaffolds were mapped on different linkage groups or distant loci within a linkage group (Additional file 1: Figure S4, Additional file 2: Table S6). This inconsistency seemed to be caused by assembly errors in these scaffolds of the current assembly. In our view, more effort to sequence the genome of the Japanese eel is necessary to identify the order of more genomic sequences on the chromosomes based on the highly dense genetic map for this species.

### Genome evolution after the TGD

**Figure 3:**
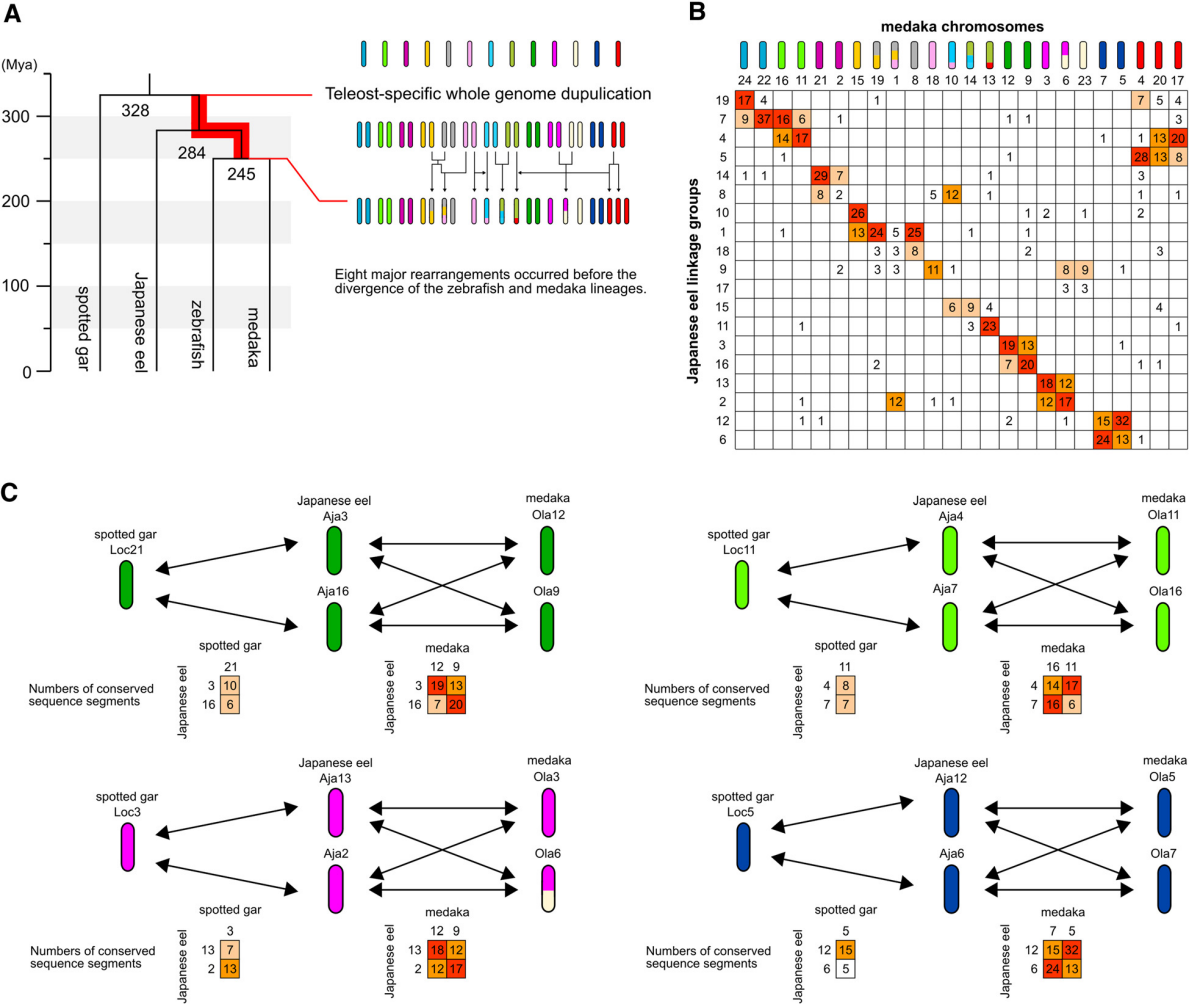
**Two-to-two correspondences between the Japanese eel and medaka chromosomes. (A)** Teleost-specific whole genome duplication (TGD) and subsequent major chromosomal rearrangement events. Kasahara et al. [19] described the estimates of the teleost ancestral chromosomes, labeled from A to M, and the eight chromosomal rearrangements. Phylogenetic relationships among fishes and their divergence times are from Broughton et al. [55]. The eel lineage diverged from other teleost lineage soon after the TGD. **(B)** Re-arrayed the Oxford grid between the Japanese eel and medaka. According to the ancestral genomic structures of zebrafish-medaka, the medaka chromosomes are re-arrayed. **(C)** Two-to-two relationships among the ohnologous chromosomes. Eel linkage group 3 (Aja3) and Aja16 shared the conserved segments with medaka chromosome 12 (Ola12) and its paralogous chromosome of Ola9. Aja3 and Aja16 also have two-to-one relationship with the gar chromosome 21 (Loc21). Similar relationships are observed in Loc11-Aja4/Aja7-Ola11/Ola16, Loc3-Aja13/Aja2-Ola3/Ola6 and Loc5-Aja12/Aja6-Ola5/Ola7.

Teleost genomes have experienced a TGD at the base of the teleost lineage [23,24,54] and eight major chromosomal rearrangements that occurred before the divergence of the zebrafish and medaka lineages ( [19], Figure 3A). Recent phylogenetic analysis estimated the divergence times of 328 million years ago (Mya) for the gar-zebrafish and 245 Mya for zebrafish-medaka [55]; therefore, the TGD and subsequent chromosomal rearrangements are presumed to have occurred in a relatively short period of ~83 Mya. The eel lineage diverged from the other teleost lineages within this period (a divergence time of 284 Mya, [55]). Hence, comparing the genome of the Japanese eel with those of other teleosts and the spotted gar would provide an opportunity to understand the history of genome evolution soon after the TGD. The medaka genome has undergone no major chromosomal rearrangements after the divergence of the zebrafish and medaka lineages and has preserved the ancestral genomic structures of two species; therefore, the medaka genome would be useful in revealing the chromosomal rearrangement events that occurred in the basal lineage of the teleosts. In contrast, the zebrafish genomes have experienced many interchromosomal rearrangements in the zebrafish-specific lineage after separating of the medaka lineage [19,20]. Hence, we focused on the comparison of the Japanese eel and medaka genomes to clarify the details of the chromosomal rearrangement events soon after the TGD. According to the ancestral genomic structures of zebrafish and medaka, we re-arrayed the medaka chromosomes in the Oxford grid between the eel and medaka (Figure 3B). As seen in the synteny analysis, some chromosome pairs have two-to-two correspondences between two species. For instance, most of the conserved segments on eel linkage group 3 (Aja3) were assigned to medaka chromosome 12 (Ola12) and its paralogous chromosome Ola9 (Figure 3C). Similarly, Aja16 shared the conserved segments with Ola12 and Ola9. By comparing the genomes of the eel-gar, most of the gar orthologs mapped on Aja3 and Aja16 were located on the single chromosome Loc21; therefore, these linkage groups are also likely to be a paralogous chromosome pair generated by the TGD. These results suggested that the quadruplet of Aja3/Aja16-Ola12/Ola9 was a group of ohnologous chromosomes, which are derived from a single ancestral chromosome before the whole genome duplication [54,56,57]. The apparent ohnologous relationships were also observed in Aja4/Aja7-Ola16/Ola11, Aja13/Aja2-Ola3/Ola6 and Aja12/Aja6-Ola7/Ola5. Such syntenic assignments that form the two-to-two correspondences among the ohnologous chromosomes are thought to arise for the following two reasons. First, the Japanese eel and medaka were diverged from their common ancestor soon after the TGD without undergoing many nucleotide changes between paralogous chromosomes of their ancestor; therefore, the genomes of two species share relatively few nucleotide changes specific to each exact pair of orthologous chromosomes, which leads to vague orthology assignments among the ohnologous chromosomes. Consequently, the case that partial sequence similarity between orthologous chromosomes and their paralogous chromosomes is higher than that between the exact pair of orthologous chromosomes occurs more often in the comparison of the eel-medaka than that of zebrafish-medaka (Additional file 1: Figure S5). Second, previous studies of the *Hox* cluster in eel species implied that this species could have highly similar sequences between paralogous chromosome pairs. Although further study of the eel genome sequence is necessary to identify the genome-wide sequence similarity between paralogous chromosome pairs of this species, a high similarity between paralogous chromosome pairs could be responsible for the increased frequency of the assignments between orthologous chromosomes and their paralogous chromosomes.

**Figure 4:**
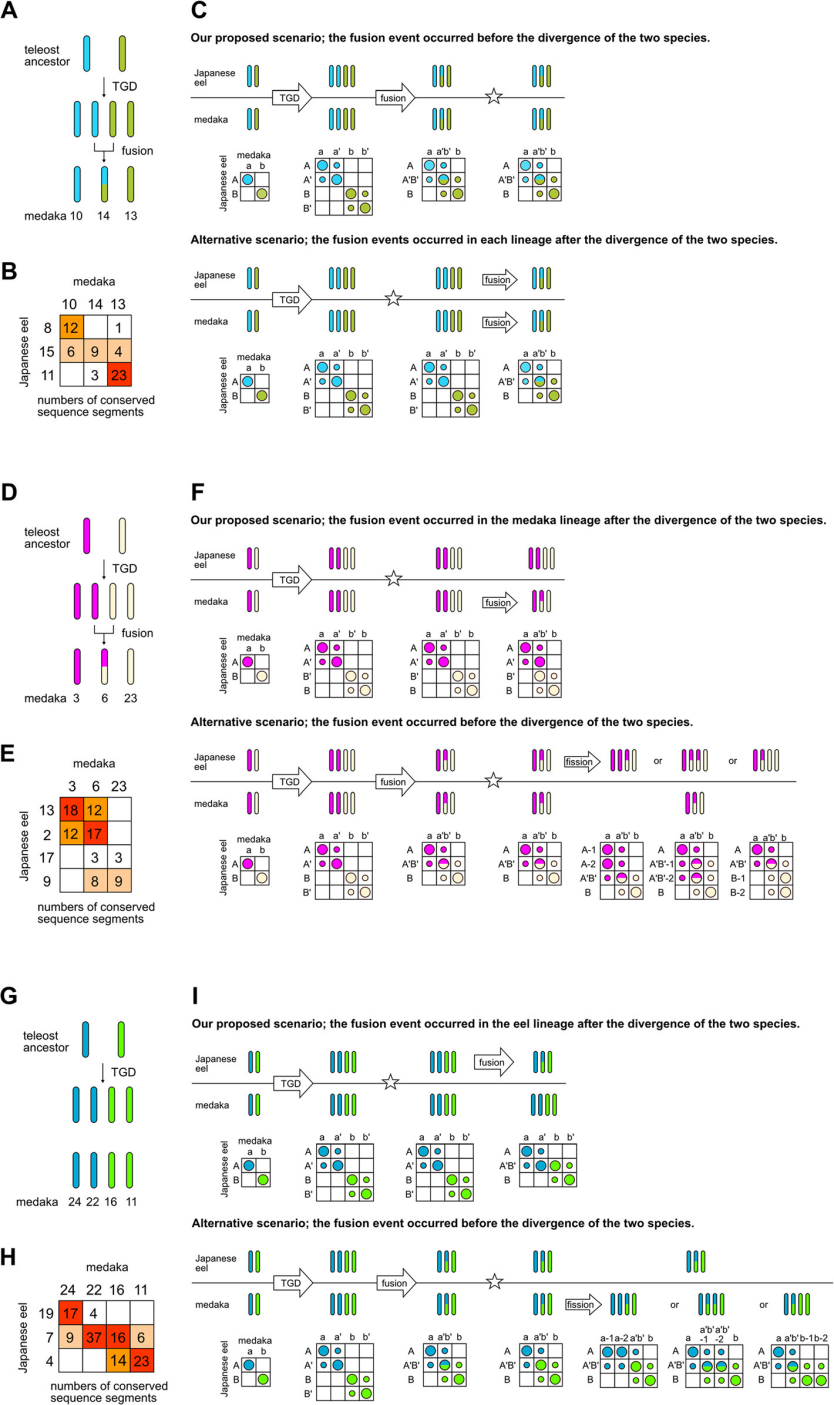
**Estimates of chromosomal rearrangements that occurred in the base of the teleost lineage. (A, D and G)** The teleost ancestral chromosomes and the chromosomal rearrangement events based on the estimates of Kasahara et al. [19]. **(B, E and H)** The Oxford grids between the Japanese eel and medaka. **(C, F and I)** Possible scenarios of the chromosomal rearrangement events and predictive models of Oxford grids between the Japanese eel and medaka for each scenario. Stars represent the divergence points of the two species. In the Oxford grids, eel chromosomes are labeled as A and B, and their duplicates are labeled as A’ and B’. Medaka chromosomes are labeled as a and b, and their duplicates are labeled as a’ and b’. Large and small circles show conserved sequence segments between orthologous chromosomes and between orthologous chromosomes and their paralogous chromosomes, respectively.

The distribution pattern of the conserved sequence segments could help us to deduce the history of genome evolution soon after the TGD. Figure 4 illustrates our proposed scenario for part of the eight major chromosomal rearrangement events at the base of the teleost lineage. Kasahara et al. [19] inferred that Ola10, Ola13 and Ola14 were derived from two ancestral chromosomes, and Ola14 was a fusion of one duplicate of the chromosomes (Figure 4A). The Oxford grid indicated that the conserved sequence segments located on these medaka chromosomes were located on eel chromosomes Aja8, Aja11 and Aja15 (Figure 4B). Given the same number of chromosomes between the two species, the eel genomes are thought to have also experienced a fusion event. In this case, two scenarios are generally conceivable (Figure 4C). One scenario is that the fusion event occurred in the common ancestral lineage before the divergence of the two species. The alternative scenario is that two fusion events occurred in each lineage after the divergence of the two species. In both scenarios, the predictive distribution of conserved sequence segments in the Oxford grid would eventually become the same pattern (Figure 4C). Indeed, the distribution of conserved segments in the actual Oxford grid was remarkably similar to that in the predictive model, even thought no segments were detected between Aja8 and Ola14. We assume that the first scenario is more likely because it is more parsimonious compared with the alternative scenario. Likewise, Kasahara et al. [19] deduced that Ola3, Ola6 and Ola23 originated from two ancestral chromosomes, and Ola6 was the fusion of one duplicate of the chromosomes (Figure 4D). These chromosomes have the correspondences to Aja2 Aja9, Aja13 and Aja17 (Figure 4E). We estimated that the four eel chromosomes have undergone no major chromosomal rearrangements after the TGD, and the fusion occurred in the medaka lineage after the divergence of the two species, because the distribution of the conserved sequence segments in the actual Oxford grid was coincident with the predictive model for this scenario (Figure 4F). An alternative scenario is that the fusion occurred in the common ancestral lineage of the two species, and the additional fission occurred in the eel-specific lineage. In the predictive model for the alternative fission scenario, at least one chromosome of the eel is presumed to have a one-to-three relationship to medaka chromosomes: for example, eel chromosomes with the structure A’B’, A’B’-1 and A’B’-2 (Figure 4F). Such a distribution was not observed in the actual Oxford grid. We therefore assume that the alternative fission scenario is rather roundabout and unlikely. In addition, the Oxford grid also indicated the trace of the interchromosomal rearrangement occurred in the eel-specific lineage. Ola24, Ola22, Ola16 and Ola11 originated from two ancestral chromosomes (Figure 4F) and have the relationships with Aja19 Aja7 and Aja4 (Figure 4G). Judging form the distribution of the conserved sequence segments in the Oxford grid, Aja7 was presumably generated by the fusion of one duplicate of the chromosomes in the eel-specific lineage (Figure 4H).

Taken together, given that the medaka genome has preserved the ancestral genomic structures of zebrafish-medaka [19], our results suggested that one of the eight major chromosomal rearrangements occurred in the ancestral lineage of eel-zebrafish-medaka after the TGD (~348 Mya to 284 Mya), and another fusion occurred after the divergence of the eel lineage and the ancestral lineage of zebrafish-medaka and before the divergence of the zebrafish and medaka lineages (284 Mya to 245 Mya). We were unable to infer the credible scenarios for the other chromosomal rearrangement events because of a lack of eel genomic sequences available for genome-wide comparison with other genomes. Further genome sequencing for eel species will provide opportunities for understanding further details of the genome evolution at the basal level of the teleost lineage and the eel-specific lineage.

## Conclusions

We constructed a second-generation linkage map of the Japanese eel using the ddRAD-seq approach with the Ion PGM sequencing platform. The maps for female and male spanned 1748.8 cM and 1294.5 cM, respectively, and were arranged into 19 linkage groups. Of the current genome assembly of the Japanese eel, 1,252 scaffolds covering 151 Mb were assigned to the 19 linkage groups using 2,260 SNP markers and 82 SSR markers. The integration of the linkage map and the assembled sequences represents an important resource for future QTL studies of this species. Comparisons of the genomes between the Japanese eel and model fishes demonstrated highly conserved synteny among teleosts and revealed part of the genome evolution soon after the TGD.

### Availability of supporting data

The ddRAD-seq reads generated by the Ion PGM platform have been deposited in the DDBJ Sequence Reads Archive (DRA) [DDBJ:DRA001739 in http://trace.ddbj.nig.ac.jp/DRASearch/].

The whole-genome shotgun reads generated by the Roche 454 GS FLX Titanium platform have been deposited in the DRA [DDBJ:DRA001189 in http://trace.ddbj.nig.ac.jp/DRASearch/].

The primer sequences for the SSR markers have been deposited in the DDBJ [DDBJ: AB500964, AB860330-AB860372 in http://getentry.ddbj.nig.ac.jp/top-j.html].

## Competing interests

The authors declare that they have no competing interests.

## Authors’ contributions

WK, KN and AF conceived and designed the study. WK, KN, AF, YN and MY conducted the experiments. KN, YK, KG and JN maintained experimental fishes. NO, TM, AO, HT, TK and MO revised the manuscript. All authors read and approved the final version of the manuscript.

## Supplementary Material

Additional file 1: Figure S1 Adapter and primer designs for constructing the ddRAD-seq library. **Figure S2:** Female linkage map of the Japanese eel. **Figure S3:** Male linkage map of the Japanese eel. **Figure S4:** Integration of the genetic map and the assembled sequences for the Japanese eel. **Figure S5:** Oxford grid between zebrafish and medaka. **Figure S6:** Correspondence between first-generation and second-generation linkage maps of the Japanese eel. **Figure S7:** Distribution of DNA markers on each linkage group of the Japanese eel. **Figure S8:** Karyotype of the Japanese eel. Click here for file

Additional file 2: Table S1 Summary of the Ion PGM sequencing run, RAD-tags and mapped RAD-tags for ddRAD-seq in Japanese eel. **Table S2:** Summary of SNP detection. **Table S3:** List of SSR markers mapped to the linkage map of Japanese eel. **Table S4:** Genotype data for the female linkage map of Japanese eel. **Table S5:** Genotype data for the male linkage map of Japanese eel. **Table S6:** Marker information, genetic distance, anchored scaffold and their blast search results for the eel genetic map. **Table S7:** Conserved sequence segments among Japanese eel, zebrafish and medaka, and the chromosome positions of their homologs in spotted gar. **Table S8:** Number of RAD loci and the region recovery in ddRAD-seq and RAD-seq approaches. Click here for file

Additional file 3 List of reference sequences for SNP discovery. Click here for file

Additional file 4 Comparison of ddRAD-seq and RAD-seq approaches. Click here for file
