# Supplementary material for: A ddRAD-based genetic map and its integration with the genome assembly of Japanese eel (Anguilla japonica) provides insights into genome evolution after the teleost-specific genome duplication
Source: BMC Genomics. 2014 Mar 26;15:233. doi: 10.1186/1471-2164-15-233 (PMC3986909; doi:10.1186/1471-2164-15-233)
Supplement: Additional file 1: Figure S1 — Adapter and primer designs for constructing the ddRAD-seq library. Figure S2: Female linkage map of the Japanese eel. Figure S3: Male linkage map of the Japanese eel. Figure S4: Integration of the genetic map and the assembled sequences for the Japanese eel. Figure S5: Oxford grid between zebrafish and medaka. Figure S6: Correspondence between first-generation and second-generation linkage maps of the Japanese eel. Figure S7: Distribution of DNA markers on each linkage group of the Japanese eel. Figure S8: Karyotype of the Japanese eel. [file 1471-2164-15-233-S1.docx]

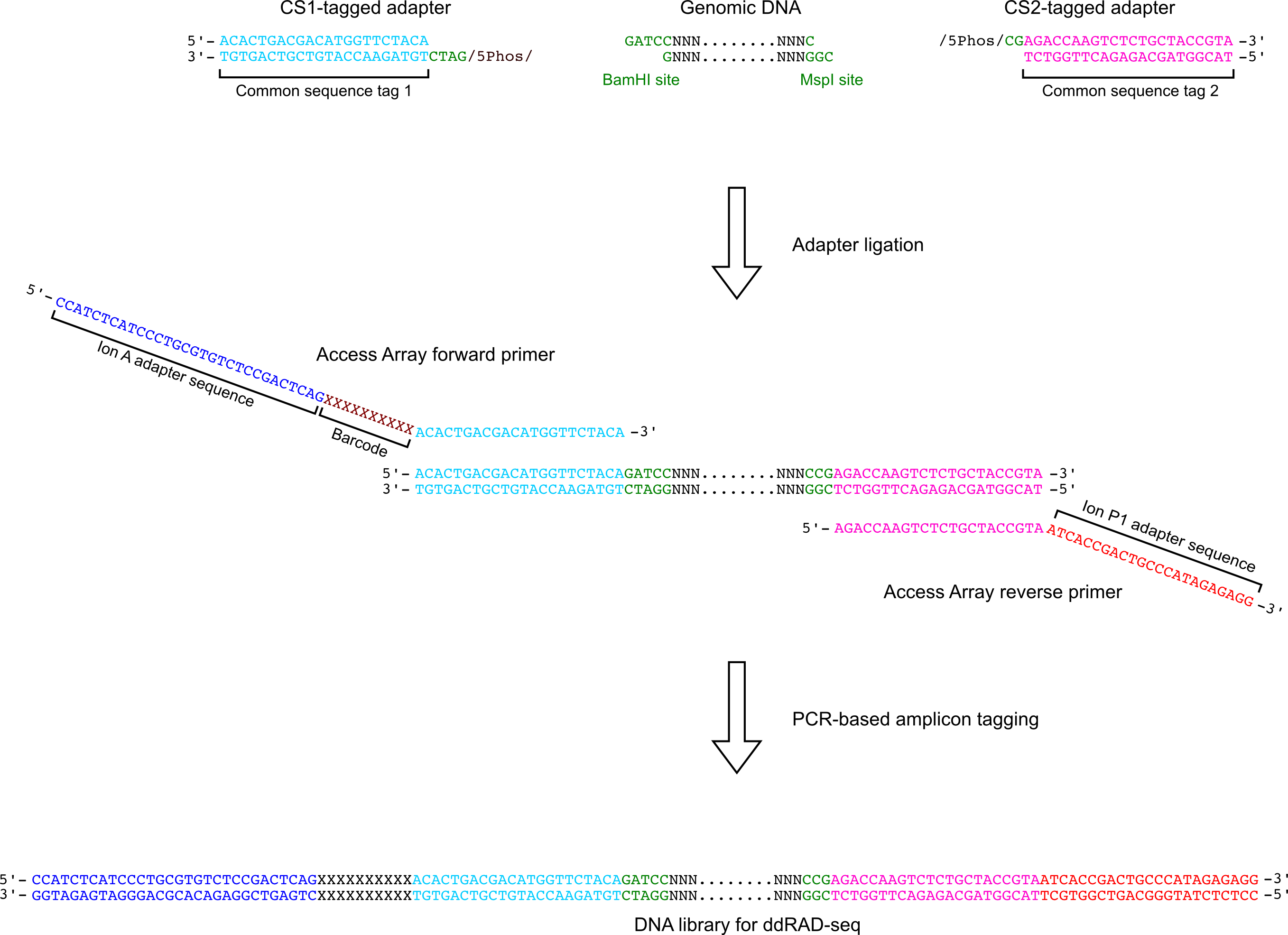


**Figure S1. Adapter and primer designs for constructing the ddRAD-seq library.** Double-digested DNA fragments are firstly ligated to the CS1-tagged adapter (cyan) and the CS2-tagged adapter (pink). The CS1 adapter binds to overhangs generated by BamHI, and the CS2 adapter contains overhangs compatible with an MspI site (green). The ligated samples are amplified with an Access Array Barcode Library for Ion Torrent PGM Sequencer-96 (Fluidigm). The forward primer consists of the Ion A adapter sequence (blue), the barcode sequences for each sample (brown) and the common sequence tag 1. The reverse primer consists of the Ion P1 adapter sequence (red) and the common sequence tag 2.


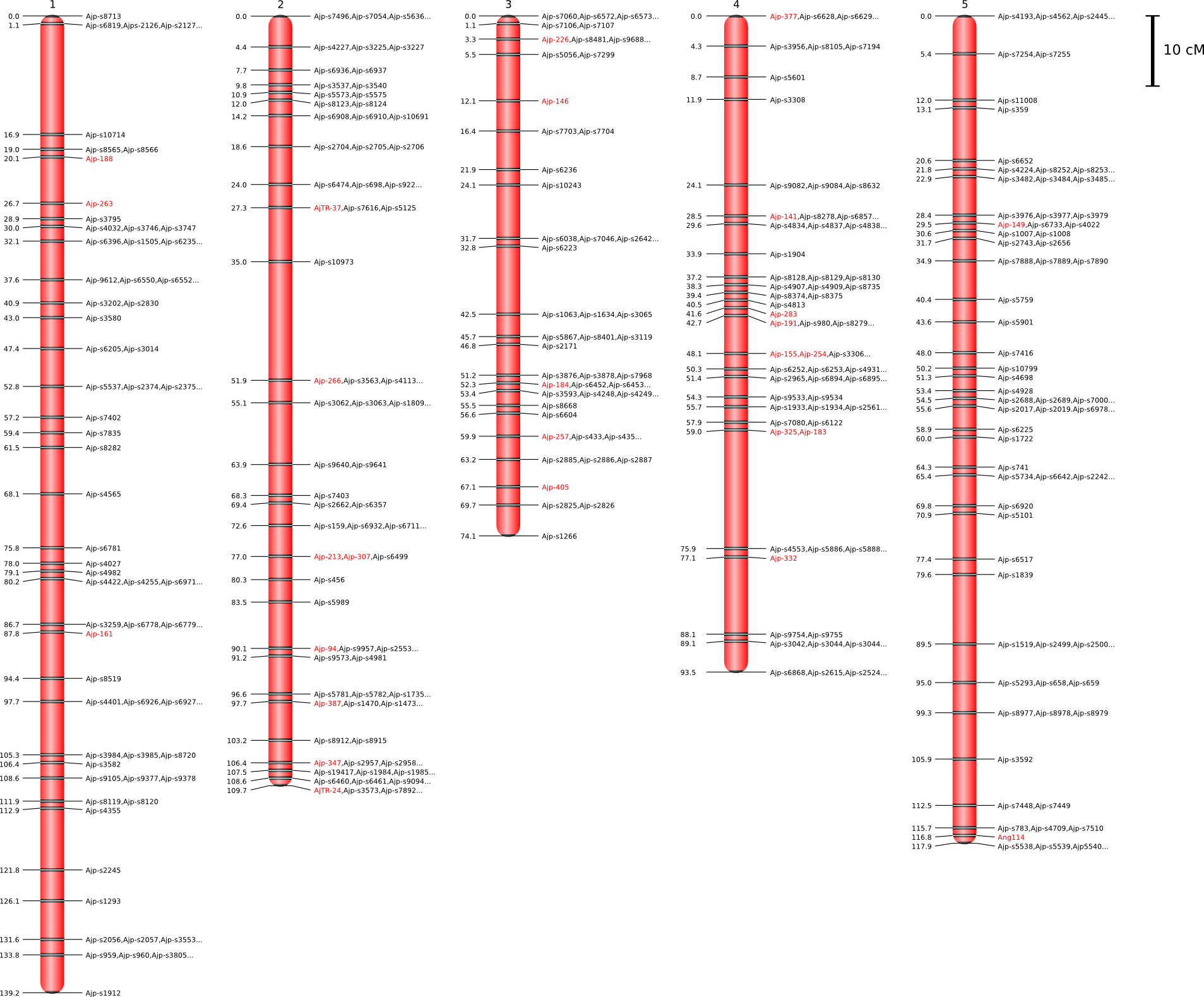


**Figure S2. Female linkage map of the Japanese eel.** Bars on each linkage group represent the loci of mapped SNP markers (black) and STR markers (red). Genetic distances (cM) were calculated using the Kosambi function. Scale bar represents 10 cM. This figure represents a part of markers mapped to linkage groups. All markers are listed in Additional file 2: Table S5.


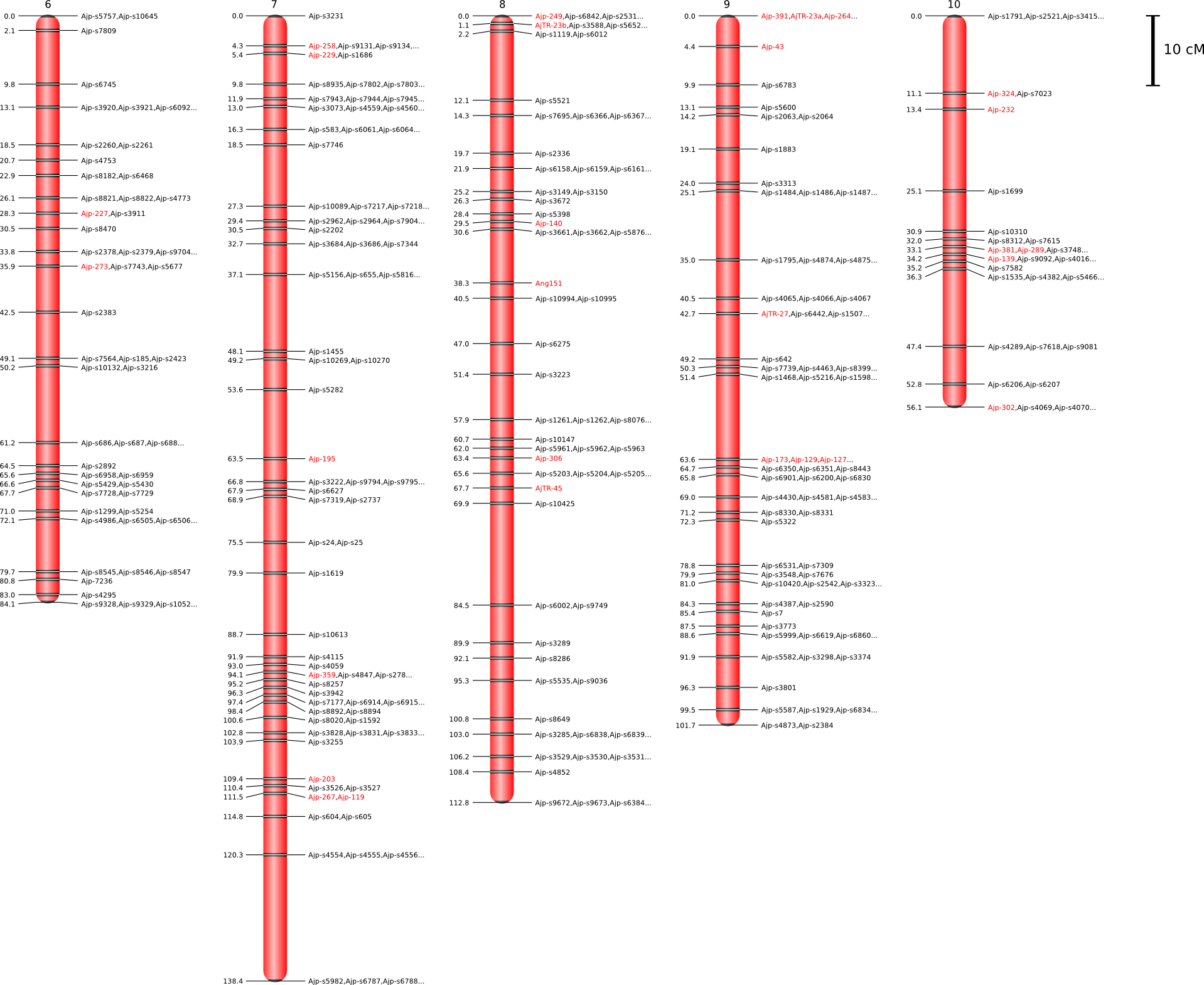


**Figure S2.** *-Continued.*


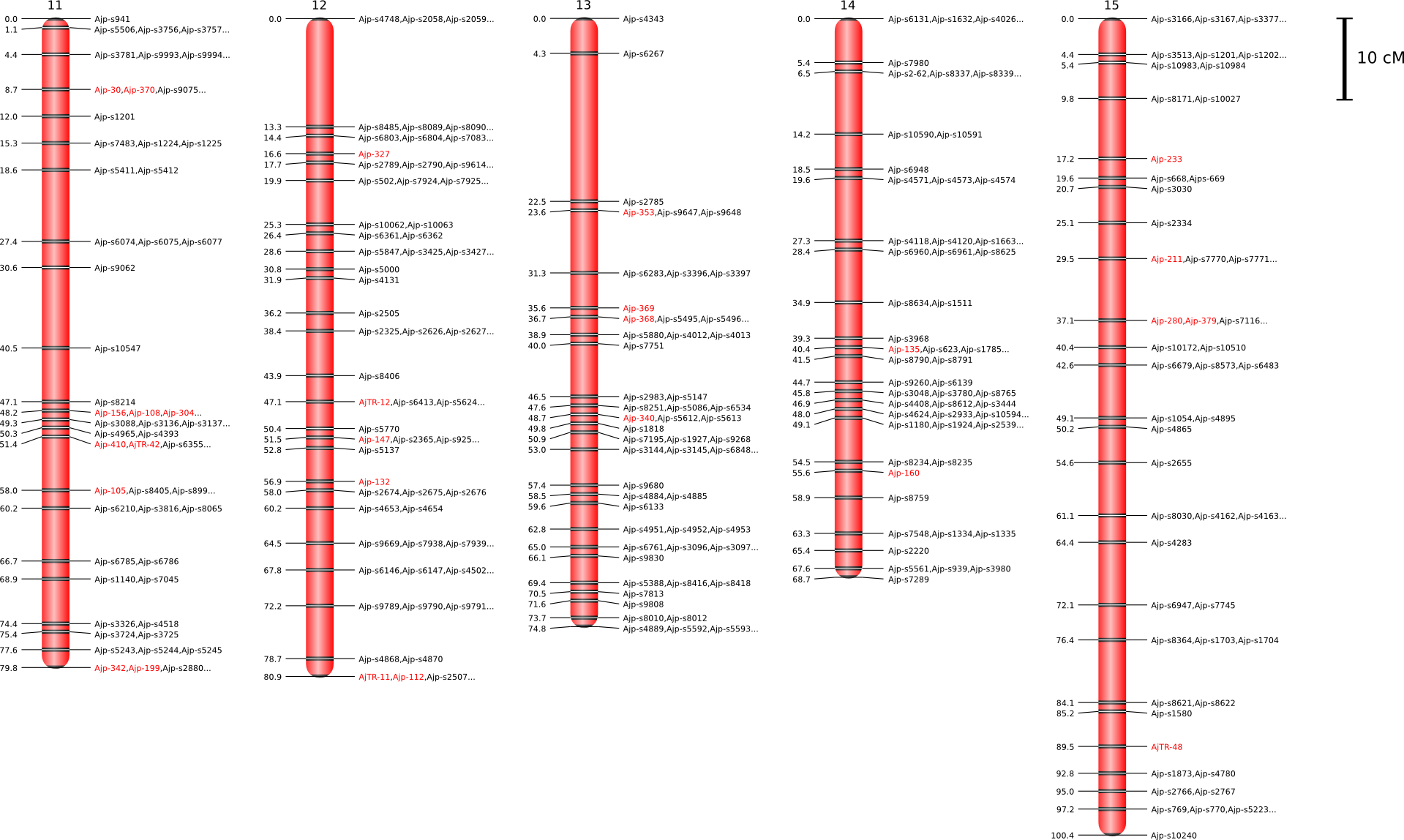


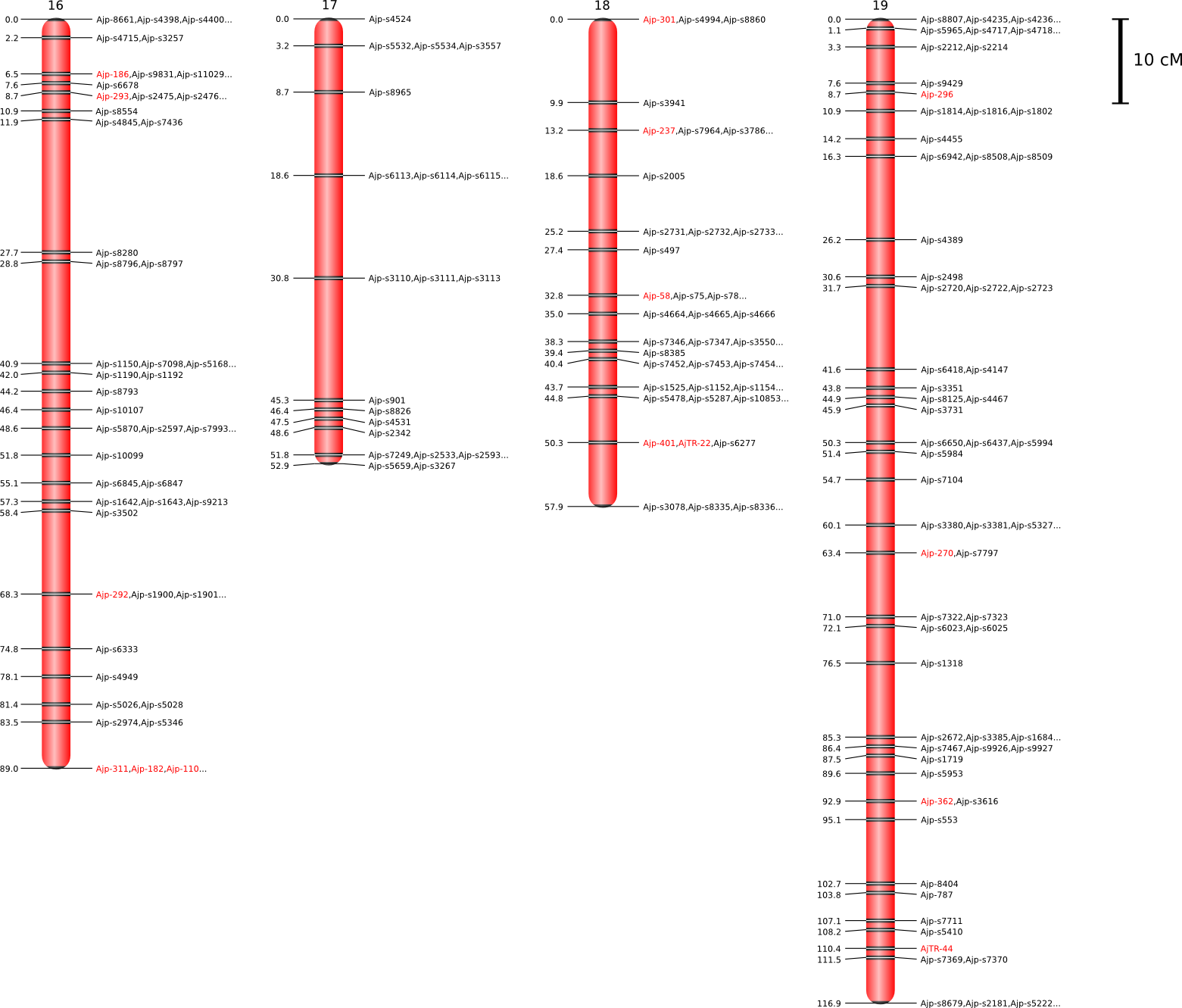


**Figure S2.** *-Continued.*


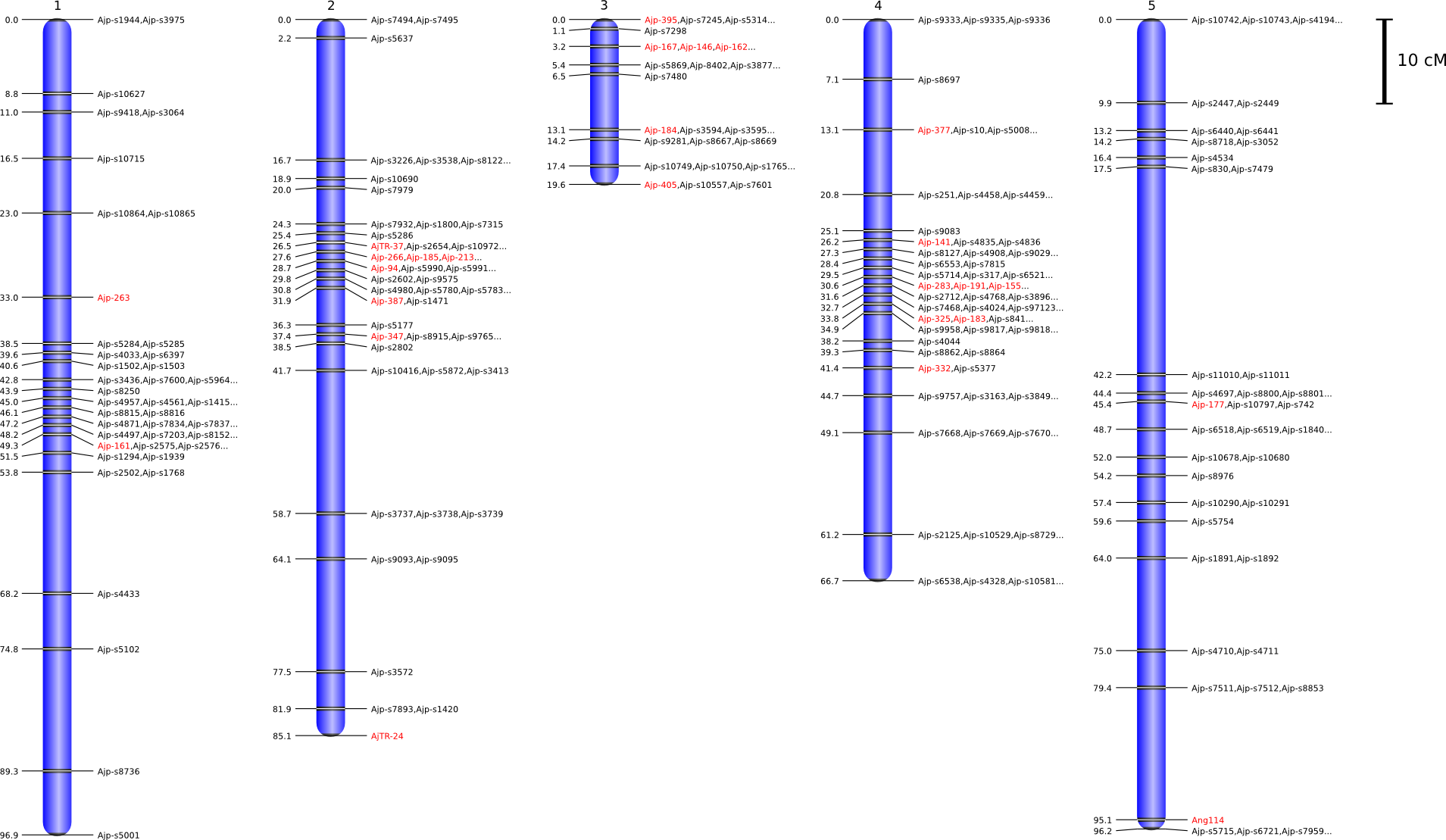


**Figure S3.** **Male linkage map of the Japanese eel.** Bars on each linkage group represent the loci of mapped SNP markers (black) and STR markers (red). Genetic distances (cM) were calculated using the Kosambi function. Scale bar represents 10 cM. This figure represents a part of markers mapped to linkage groups. All markers are listed in Additional file 2: Table S5.


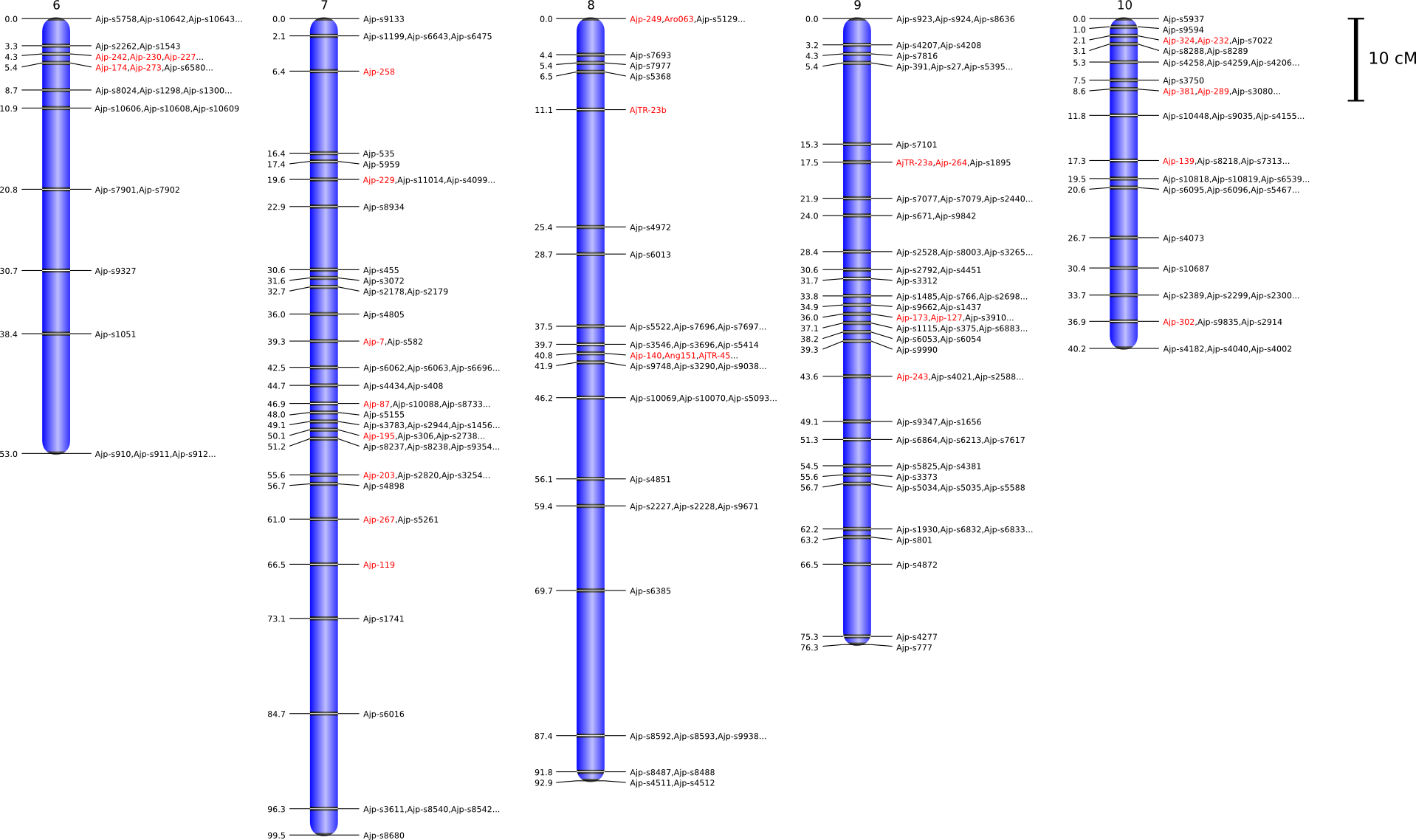


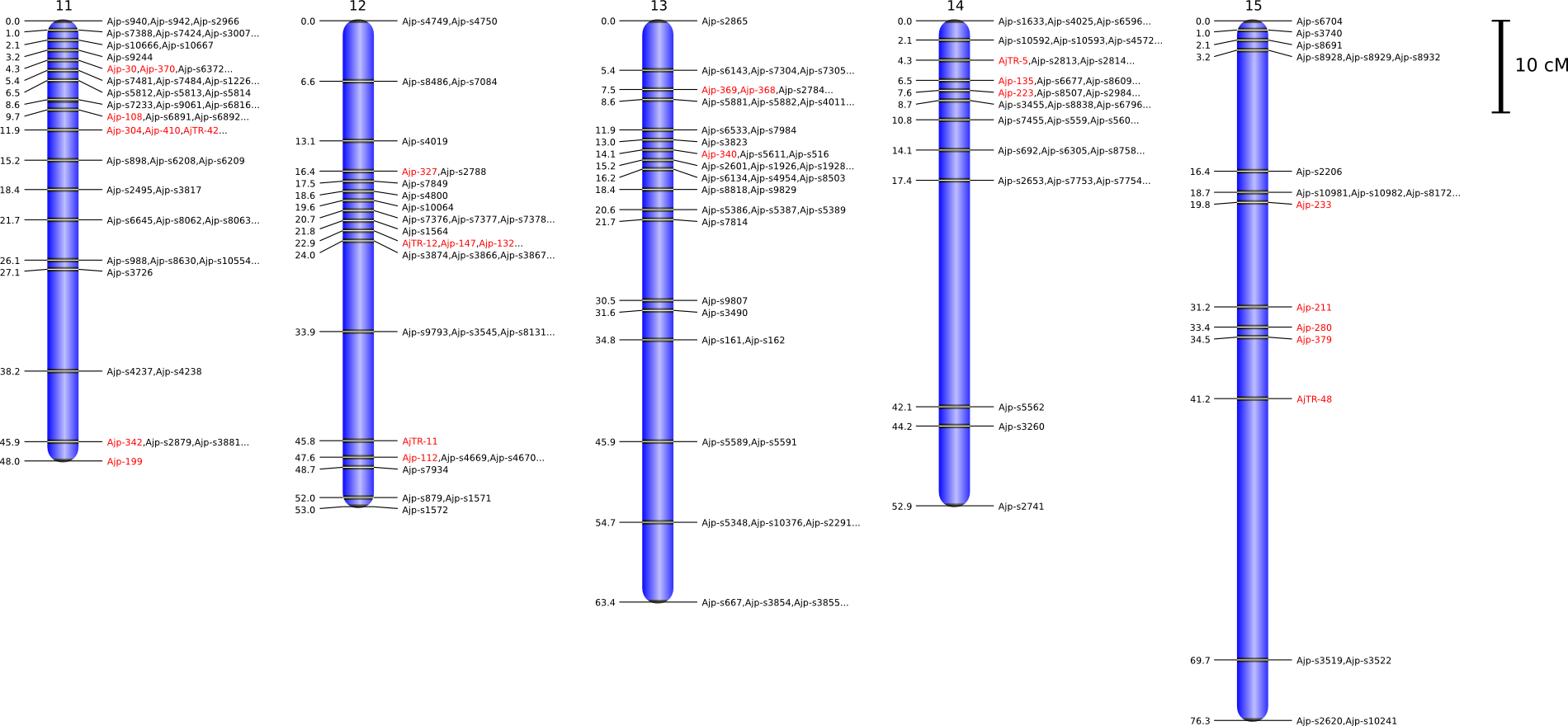


**Figure S3.** *-Continued.*


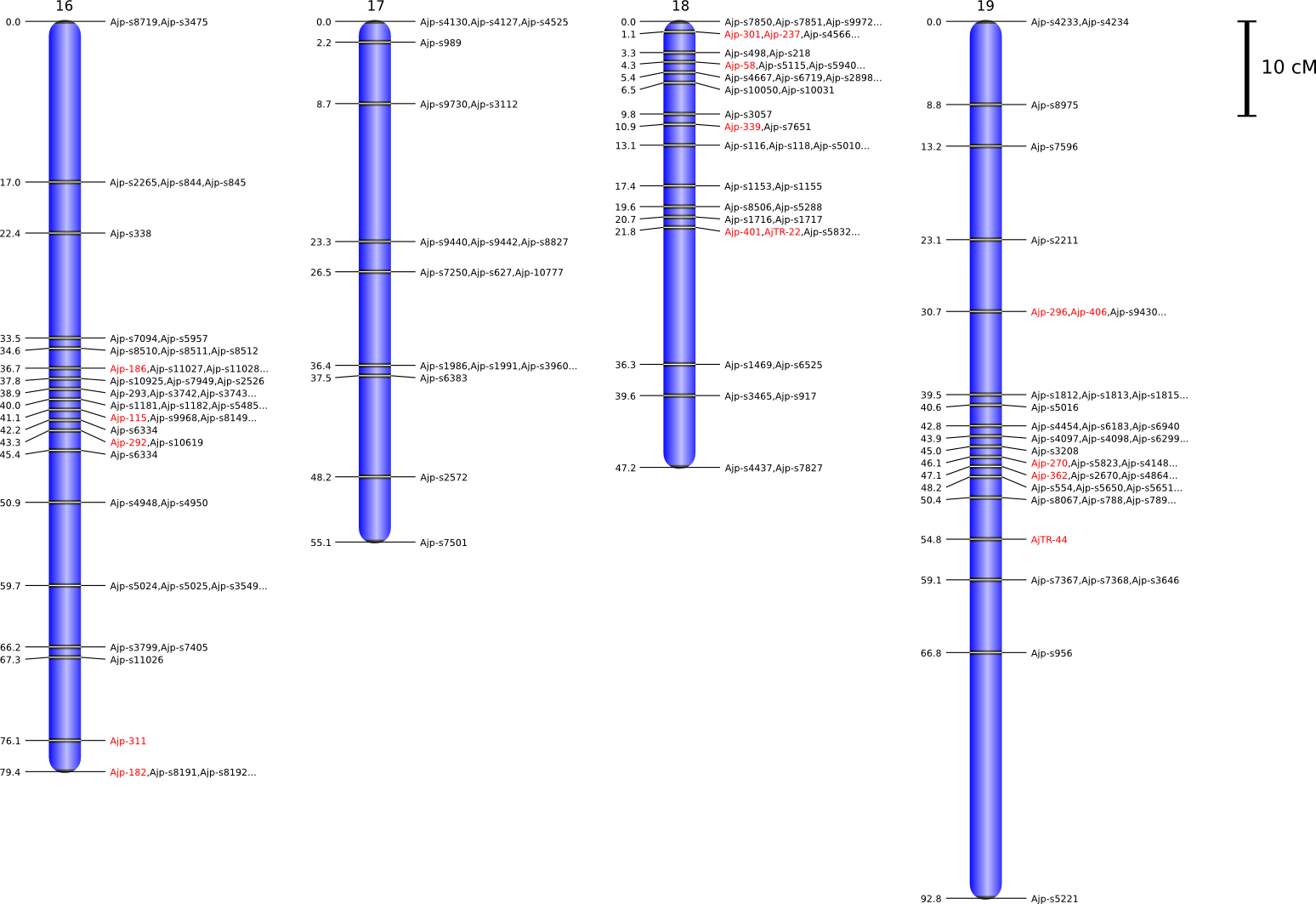


**Figure S3.** *-Continued.*


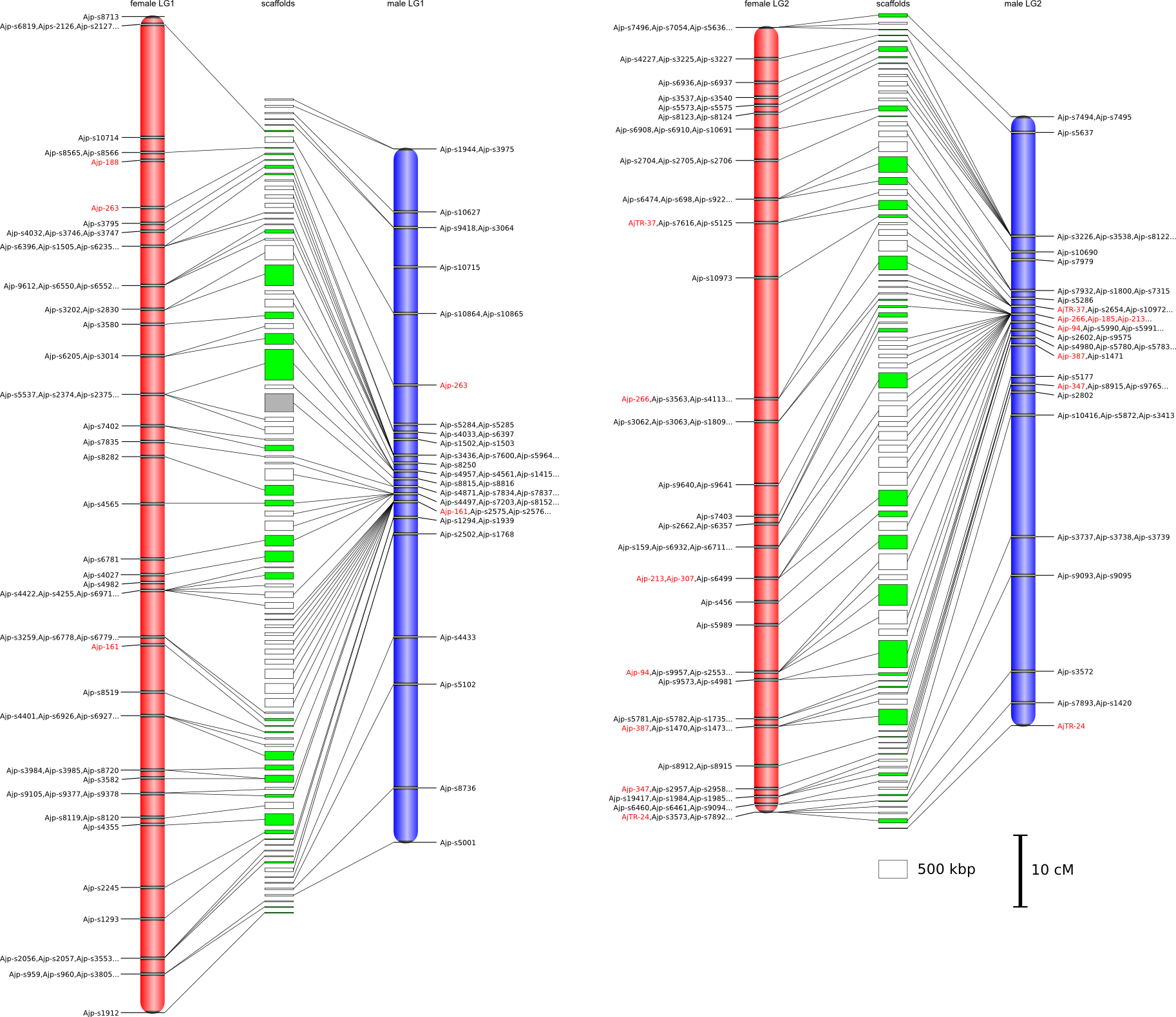


**Figure S4.** **Integration of the genetic map and the assembled sequences for the Japanese eel.** Vertical red and blue bars represent female and male linkage group, respectively. SNP markers are black and STR markers are red. Boxes sandwiched between the female and male linkage groups represent scaffolds. Scaffolds for which a relative order on each chromosome has been determined are labeled in green. Scaffolds that are mapped on other linkage groups are labeled in gray. Transverse lines link the location of each marker on the genetic maps with its corresponding anchored scaffolds. Gaps between scaffolds are arbitrary because the actual distance between scaffolds is unknown. Scale bars represent 10 cM and 500 kb.


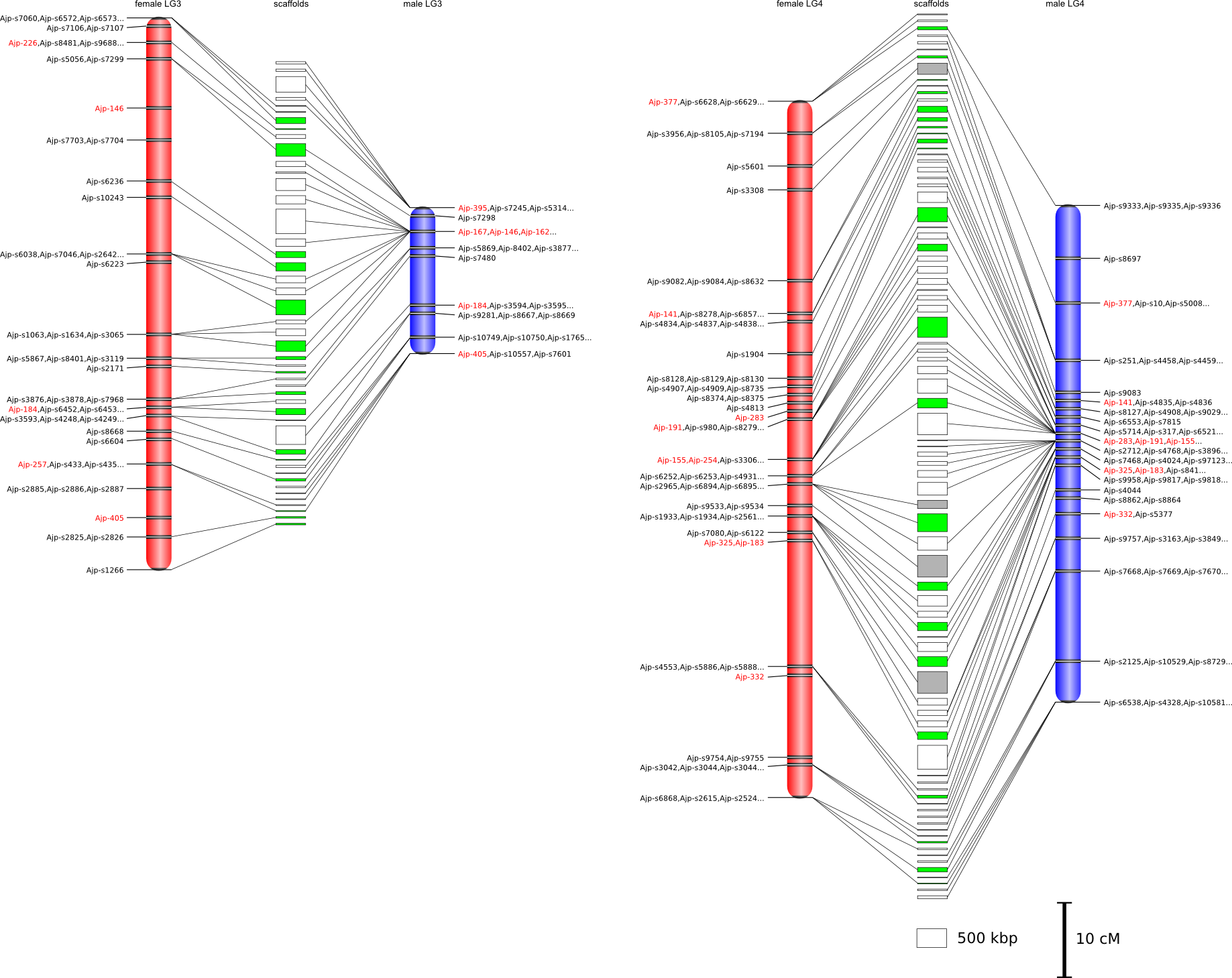


**Figure S4.** *-Continued.*


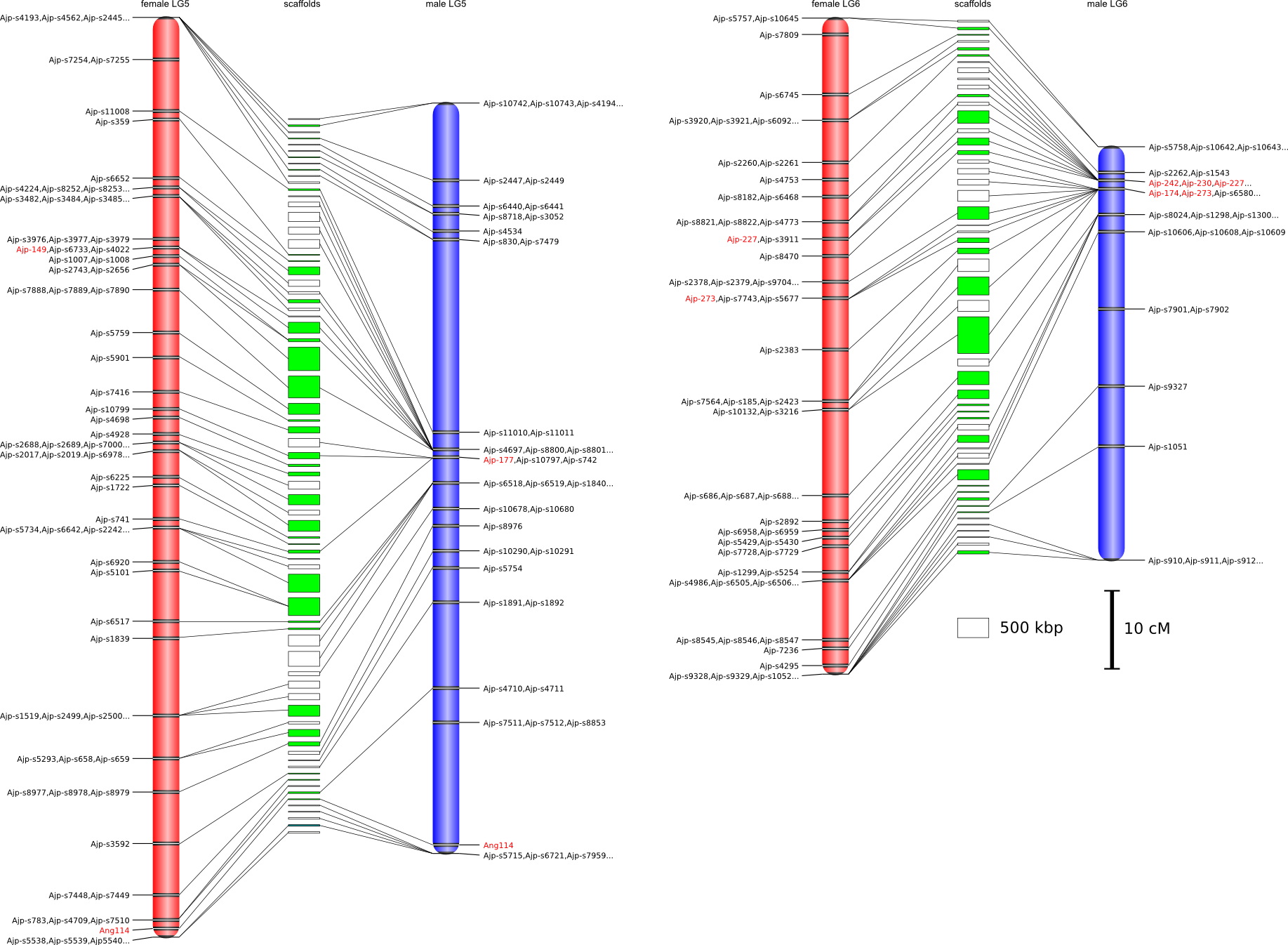


**Figure S4.** *-Continued.*


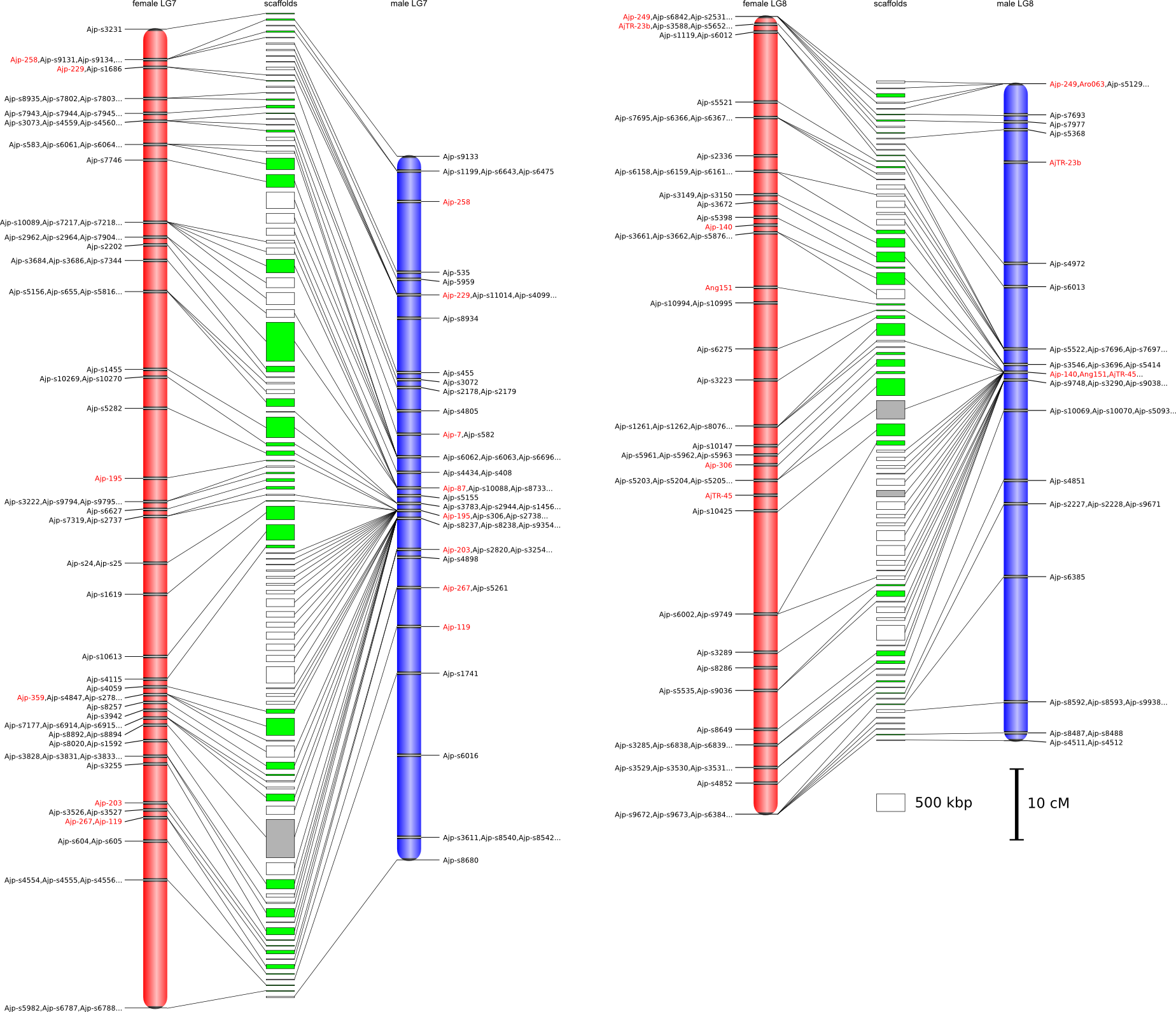


**Figure S4.** *-Continued.*


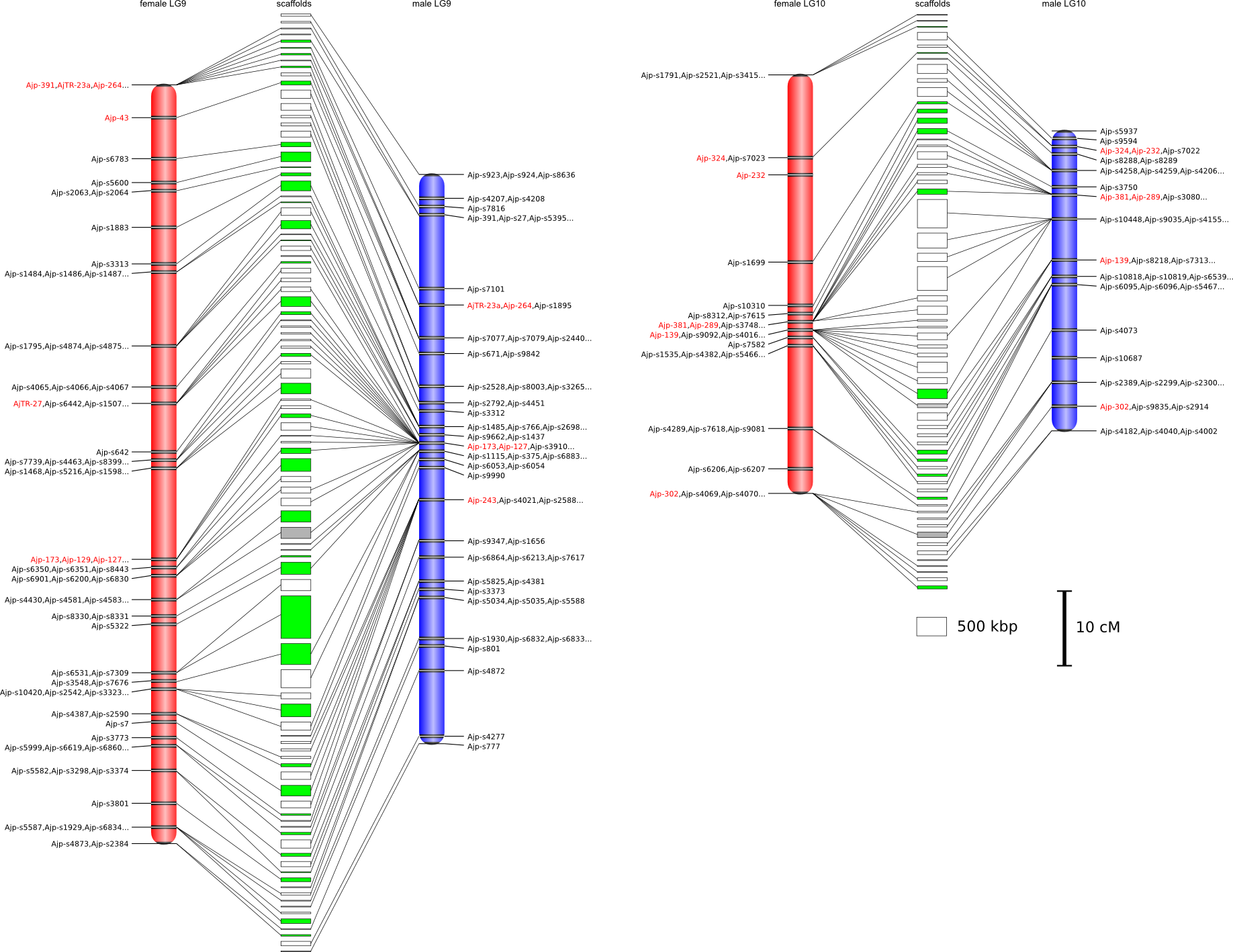


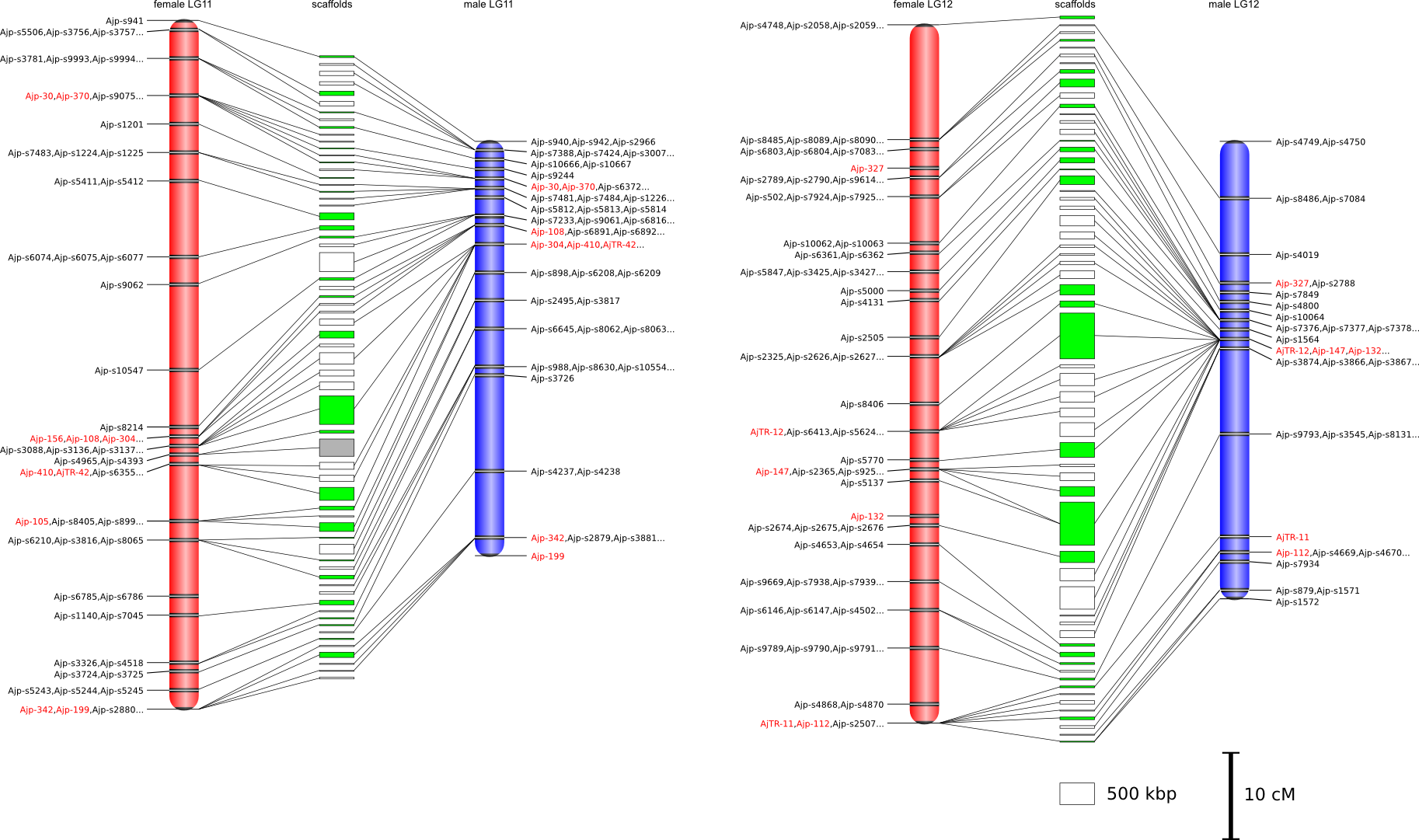


**Figure S4.** *-Continued.*


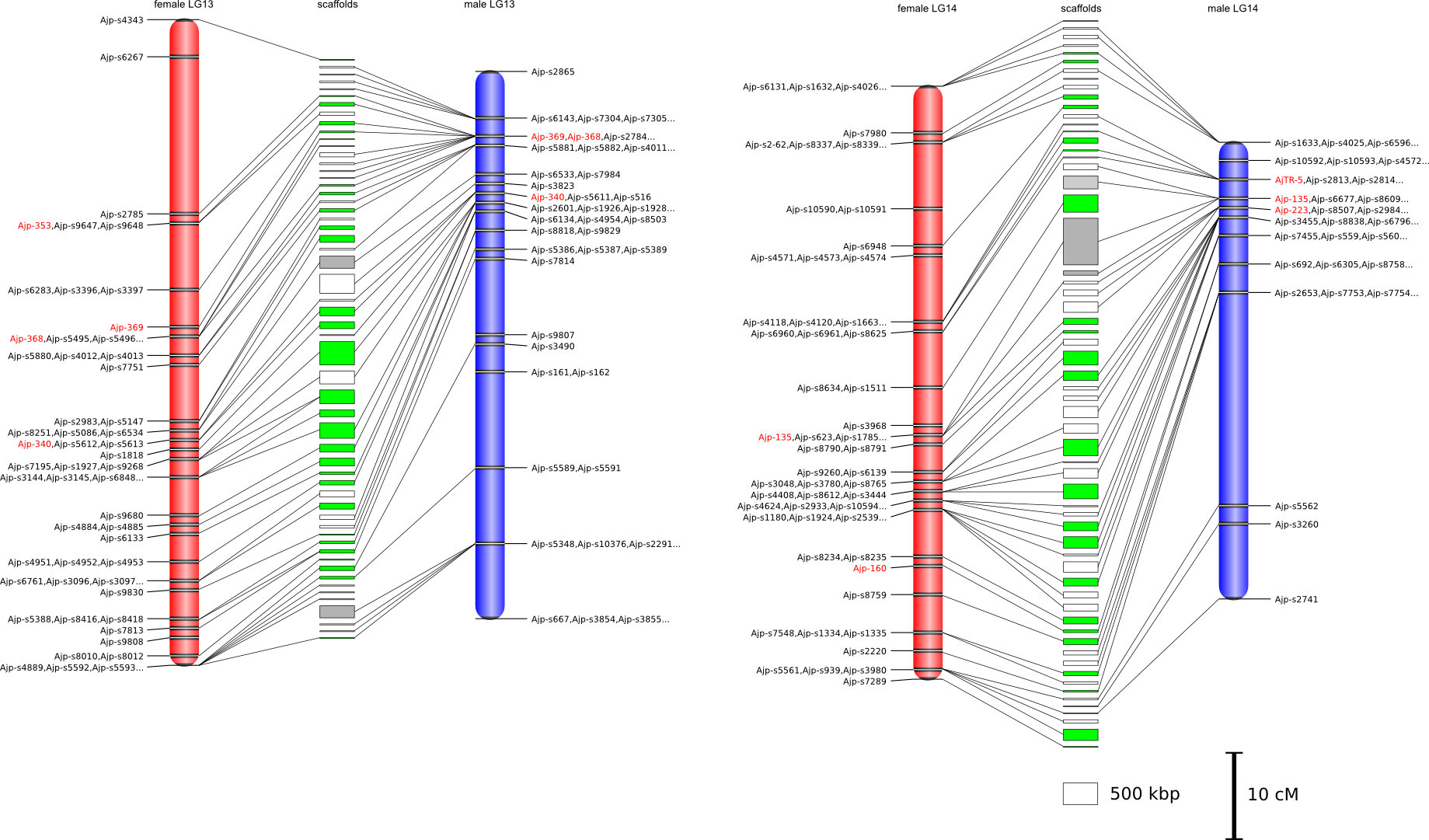


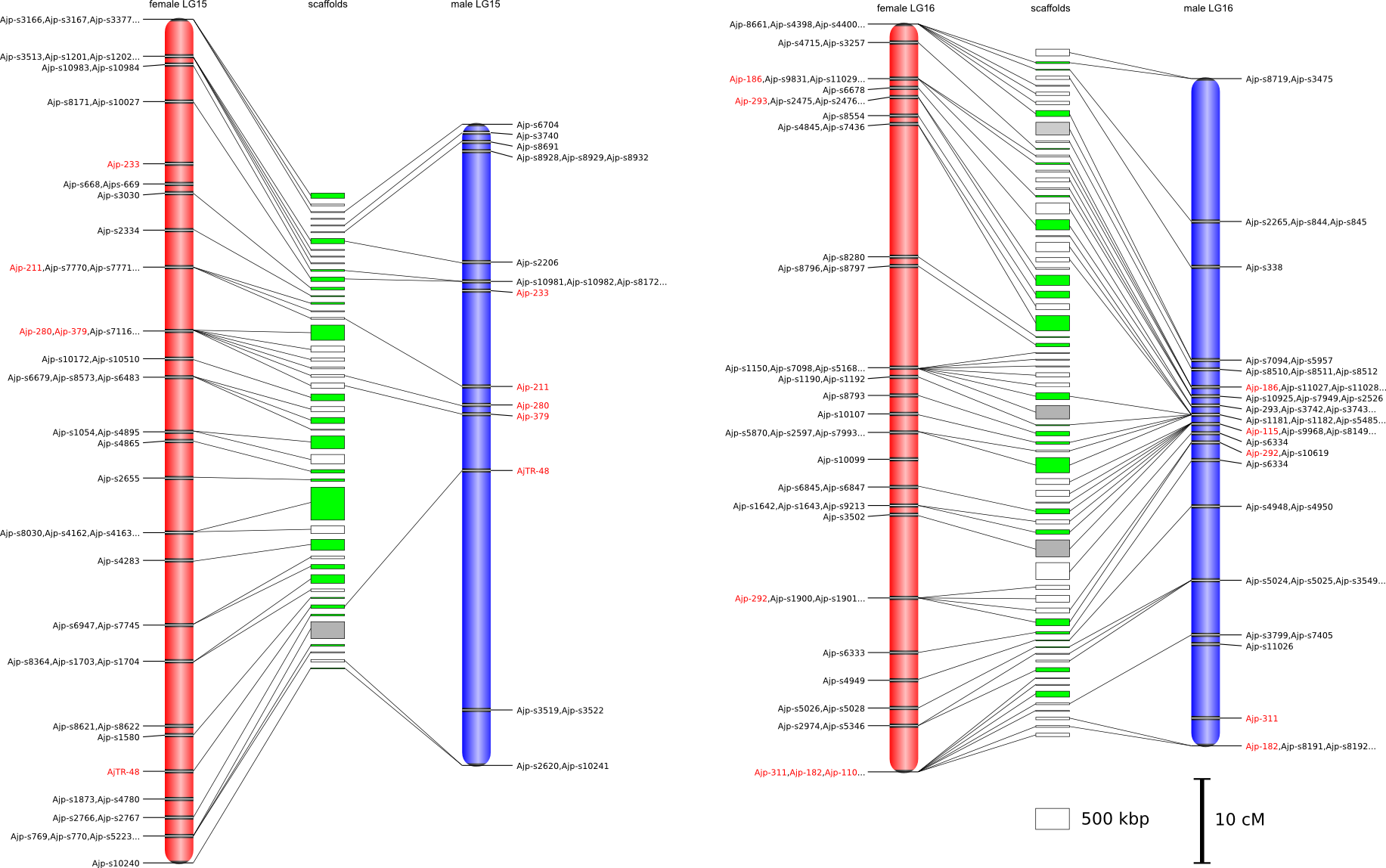


**Figure S4.** *-Continued.*


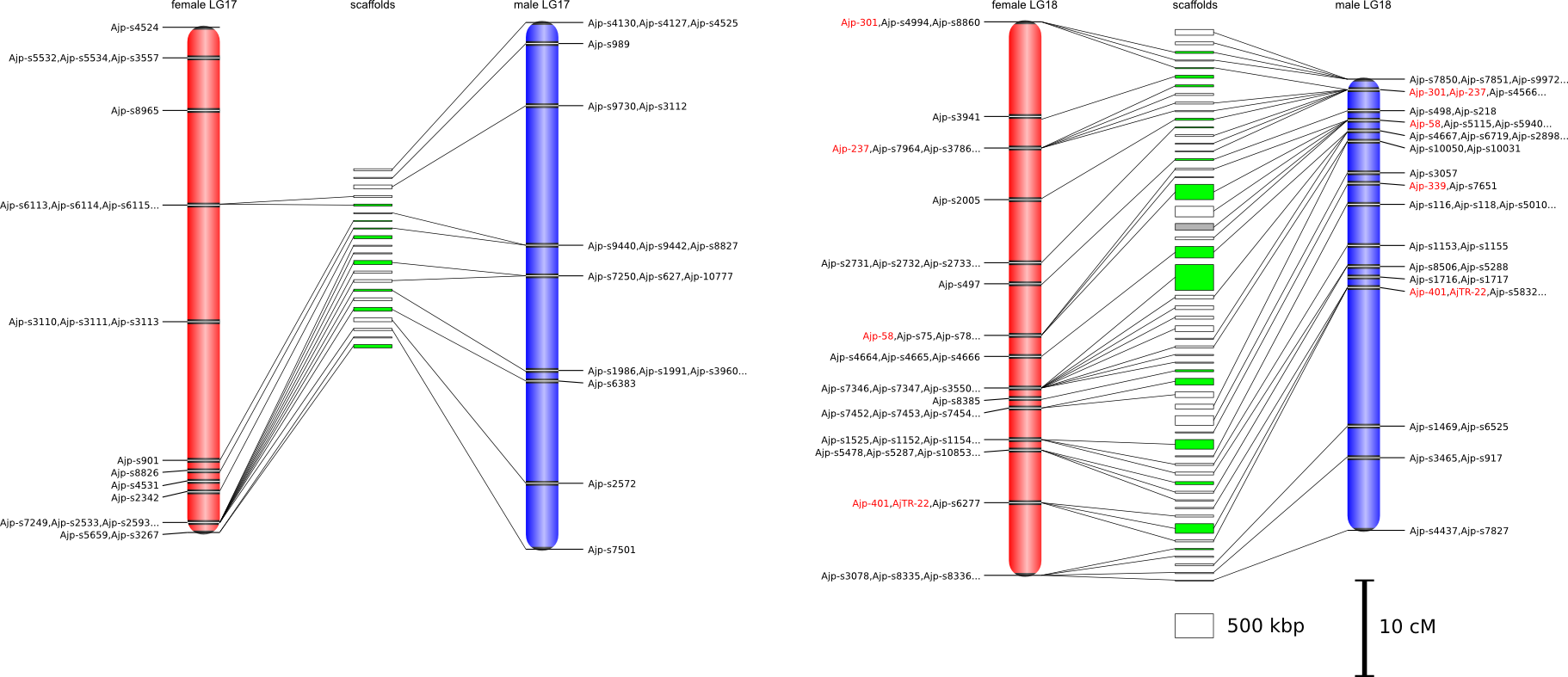


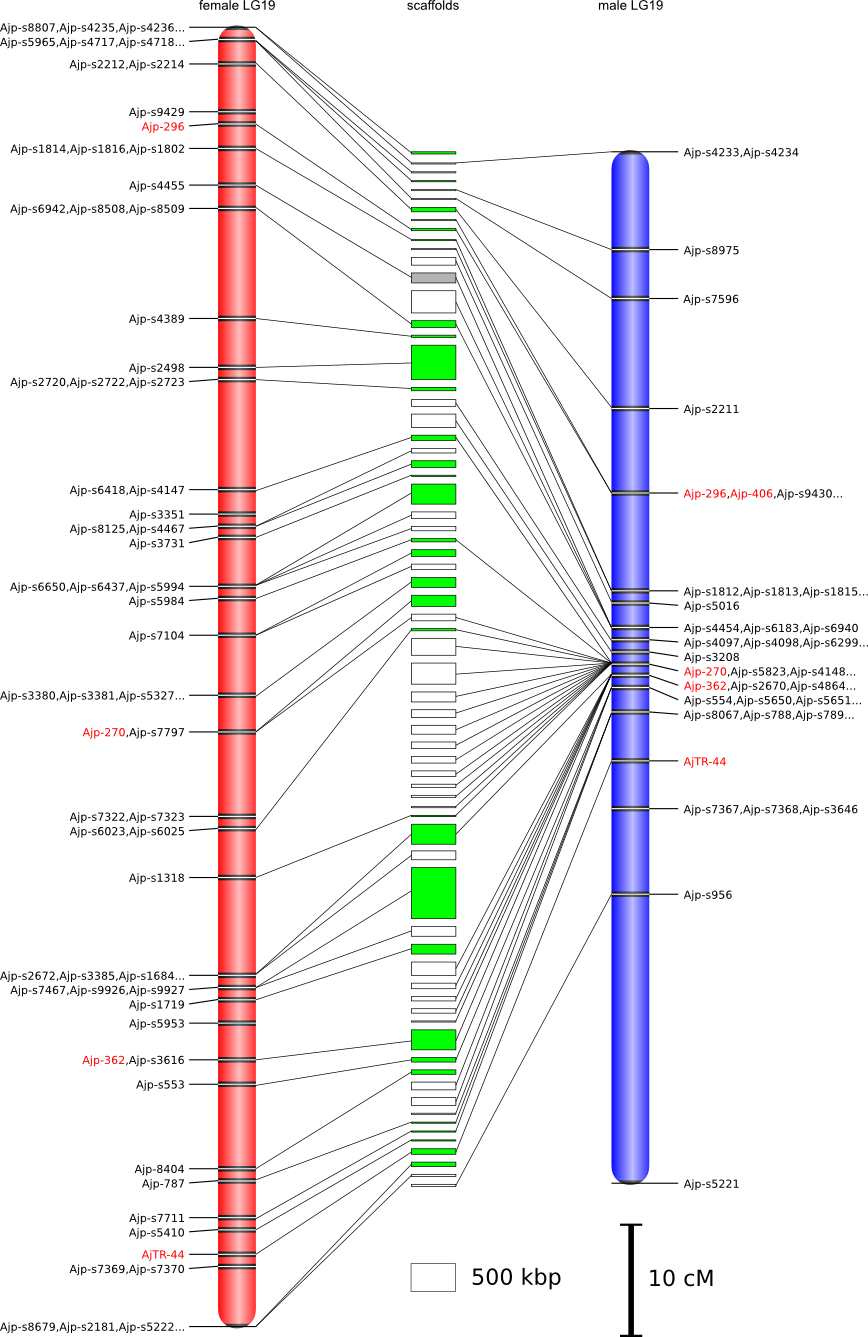


**Figure S4.** *-Continued.*


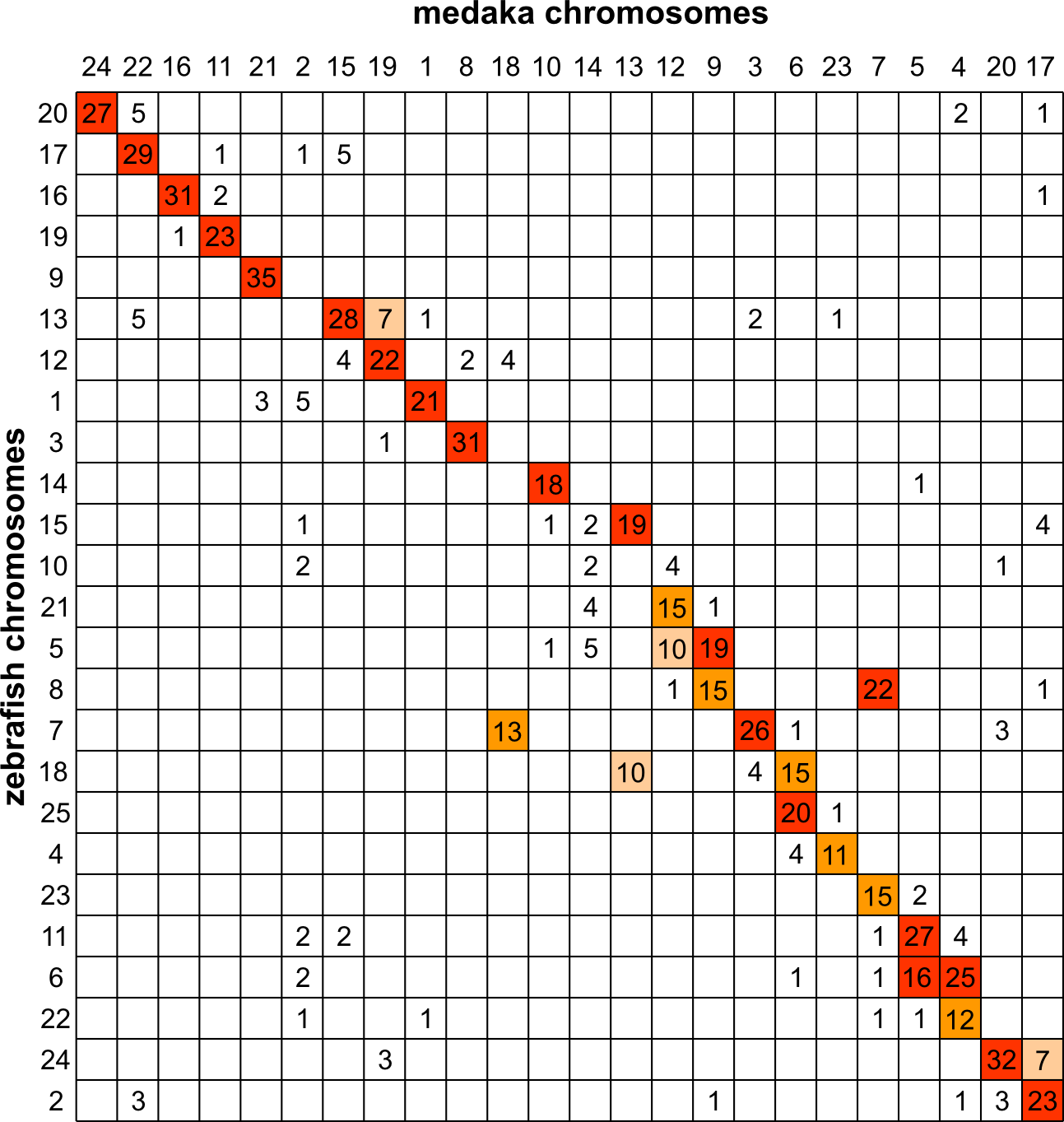


**Figure S5.** **Oxford grid between zebrafish and medaka.** Conserved sequence segments are arrayed according to chromosome for each species. Numbers in boxes indicate the number of the conserved sequence segments. Of the total segments, 624 of 745 (84%) segment pairs fell in 31 syntenic boxes, each composed of six or more segment pairs (Figure 3A). Among them, 494 (66%) segment pairs were mapped into 20 syntenic boxes that had at least 16 segment pairs, and 121 (16%) segment pairs did not fall into syntenic boxes. The details of the conserved sequence segments are listed in Additional file 2: Table S6.


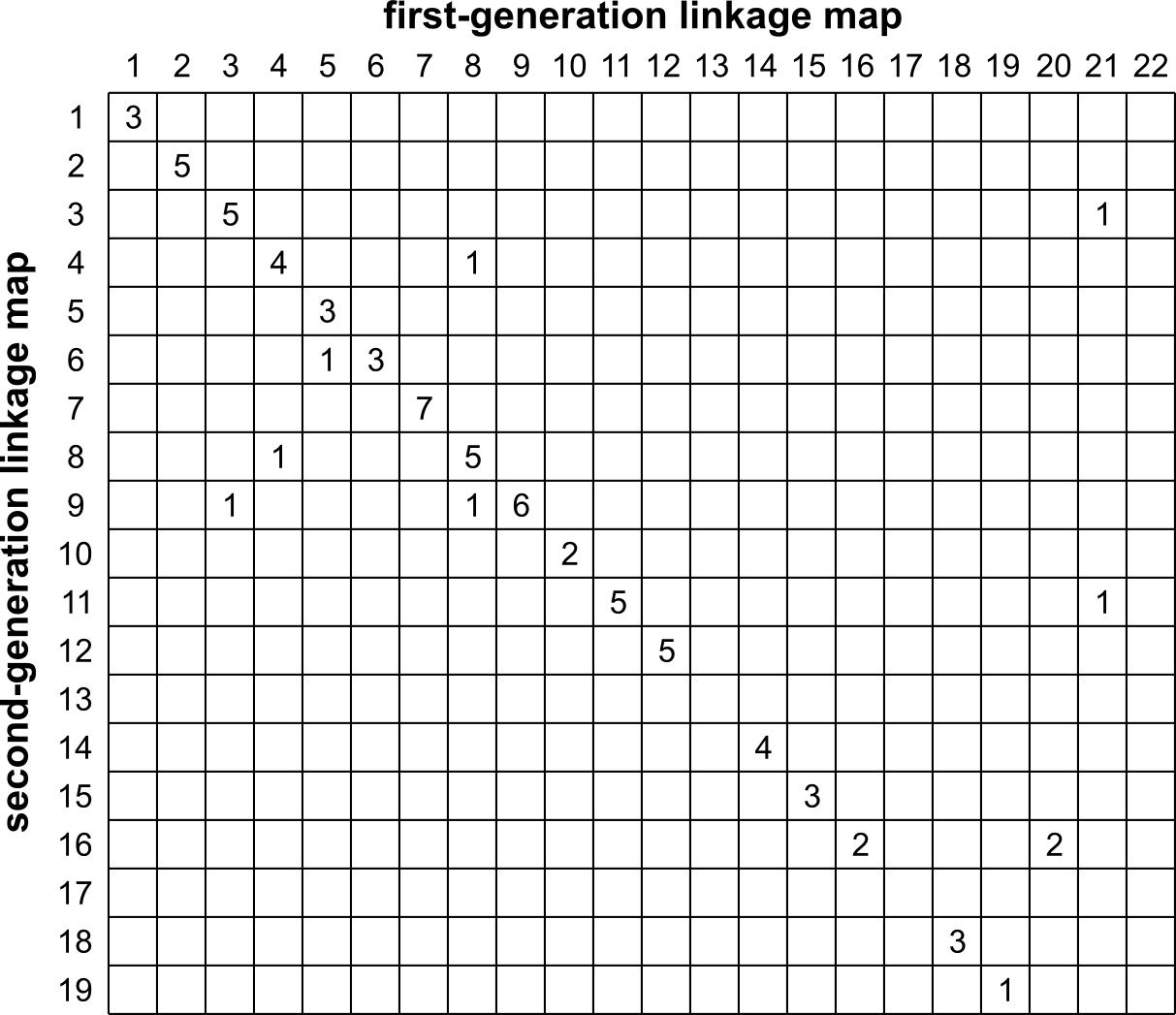


**Figure S6.** **Correspondence between first-generation and second-generation linkage maps of the Japanese eel.** STR markers are arrayed according to linkage groups for previous and present maps. Numbers in boxes indicate the number of SSR markers. Linkage group 20 (LG20) of the previous map has been integrated with LG16 in the present map. A linkage relationship between the markers located on LG21 of the previous map was disrupted, and the markers have been mapped on LG3 and LG11, respectively. The correspondences for LG13, LG17 and LG22 from the previous map are unknown because of the absence of SSR markers.

**A** male linkage groups

**
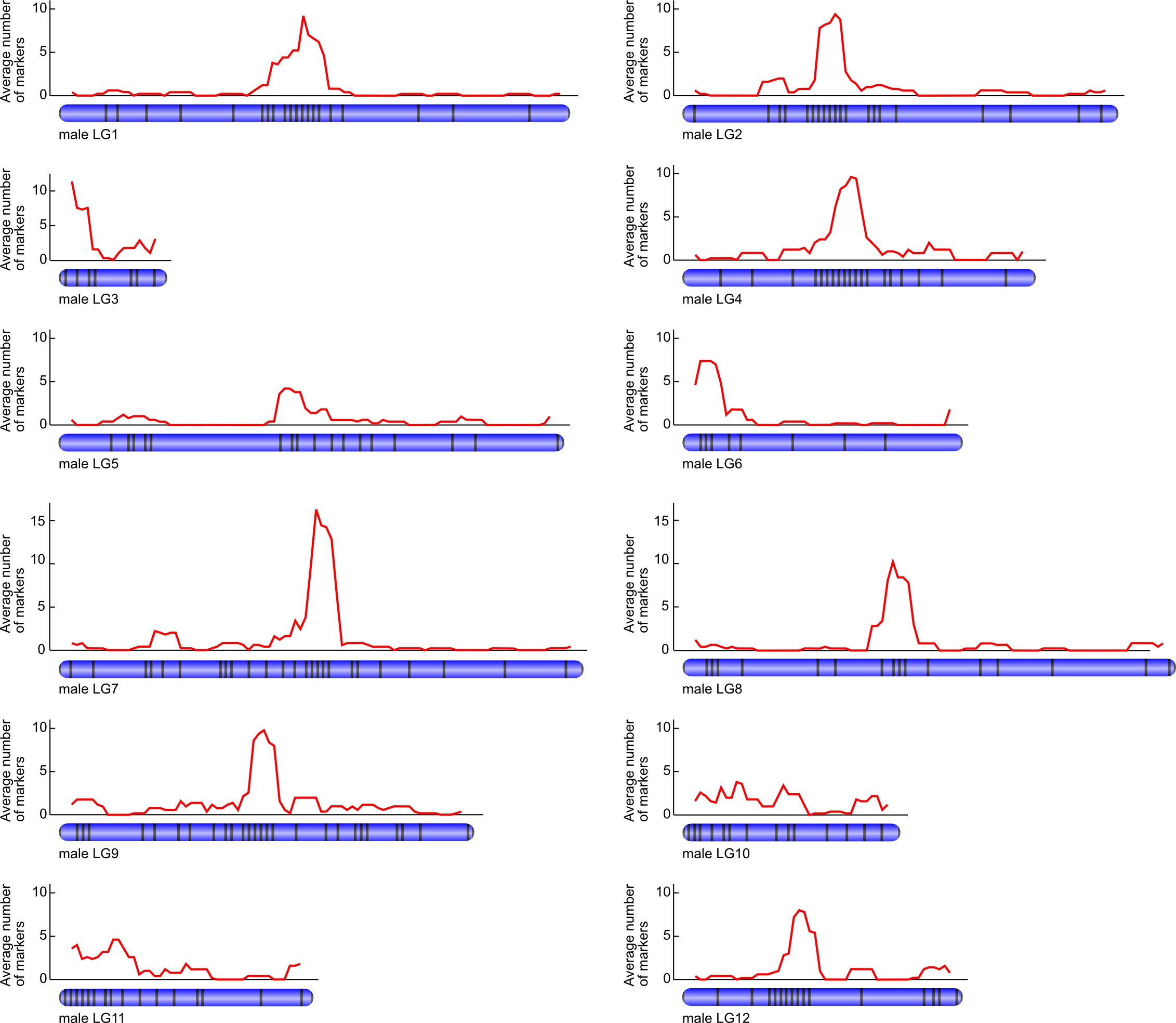
**

**Figure S7.** **Distribution of DNA markers on each linkage group of the Japanese eel.** The average number of DNA markers across each linkage group was calculated using a sliding window method with a window size of 5 cM and a sliding step of 1 cM. (**A**) Distribution of DNA markers on the male linkage map. (**B**) Distribution of DNA markers on the female linkage map. (**C**) Details of linkage group 10 and 18. Transverse lines represent the positional relationship between the markers on the female and male linkage maps.

**
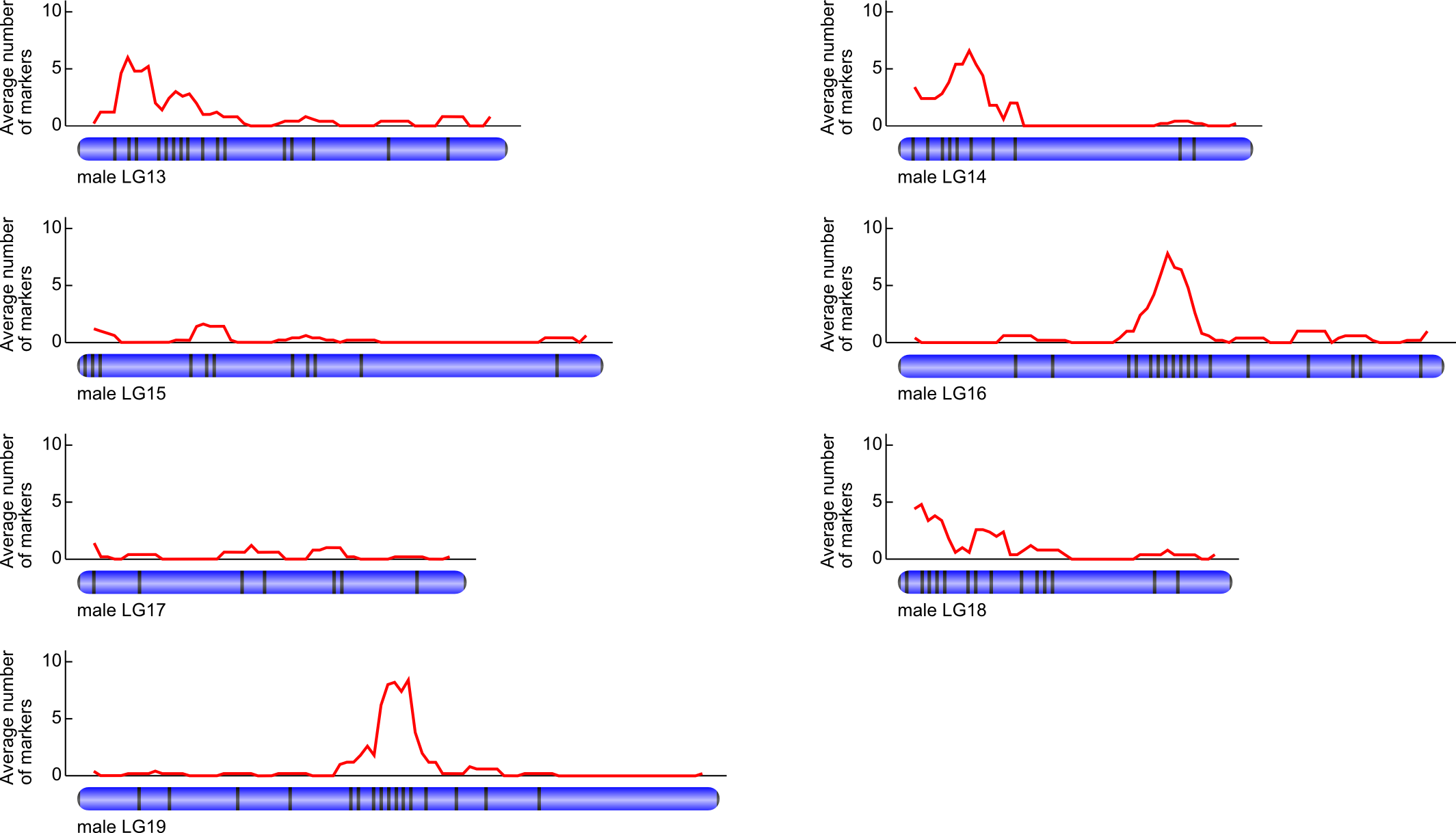
**

**Figure S7.** *-Continued.*

**B** female linkage groups


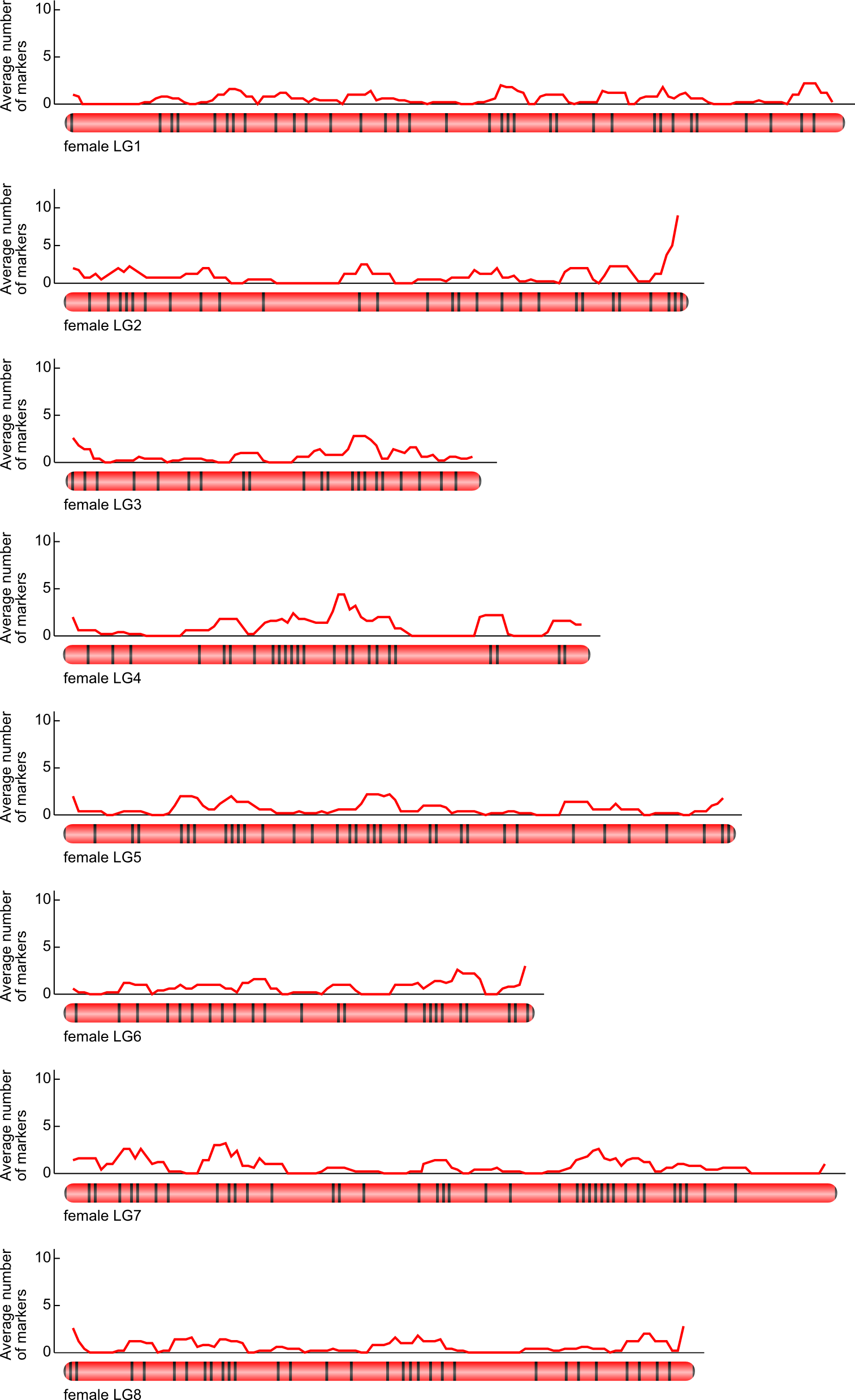


**Figure S7.** *-Continued.*

*
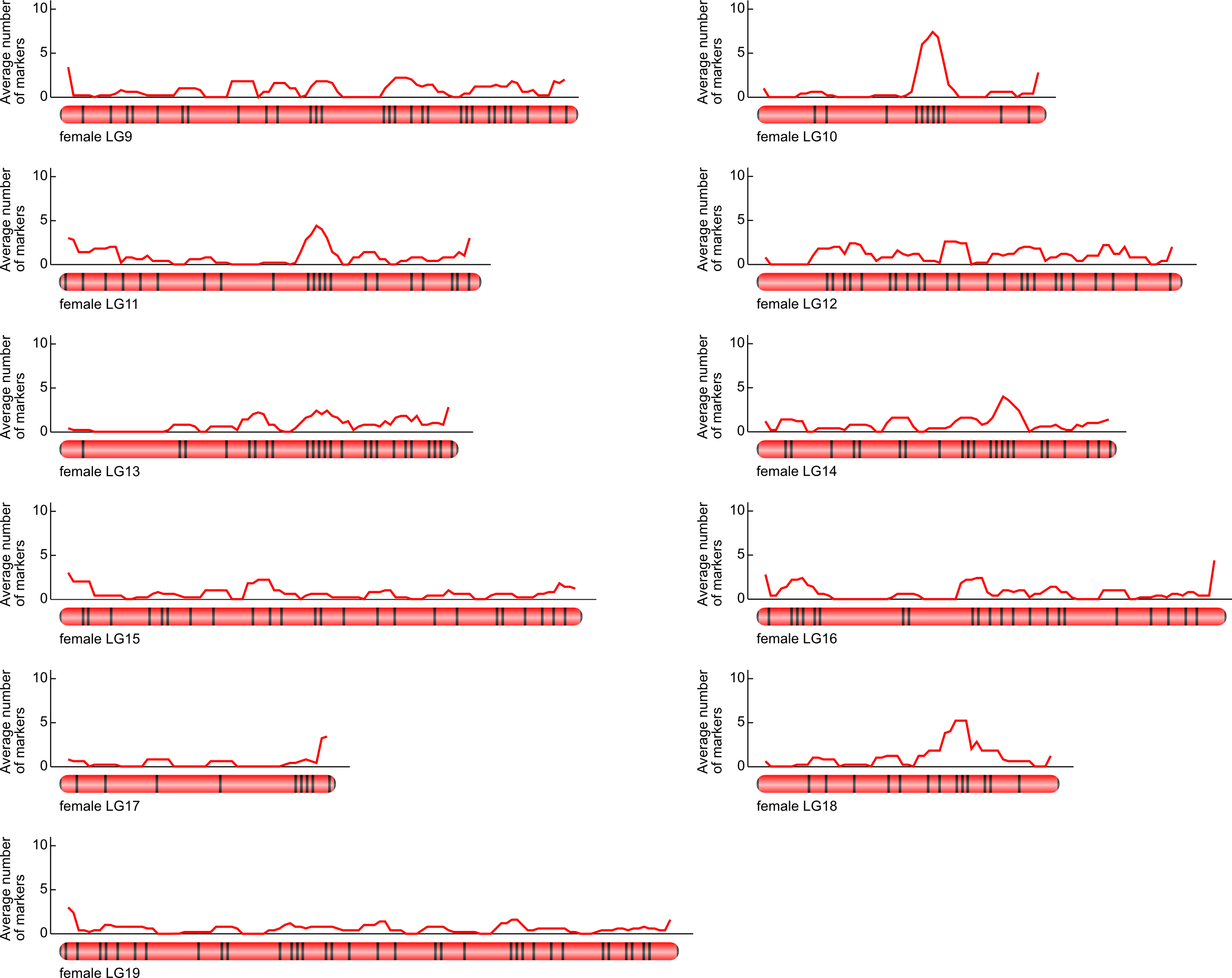
*

**C** LG10 and LG18


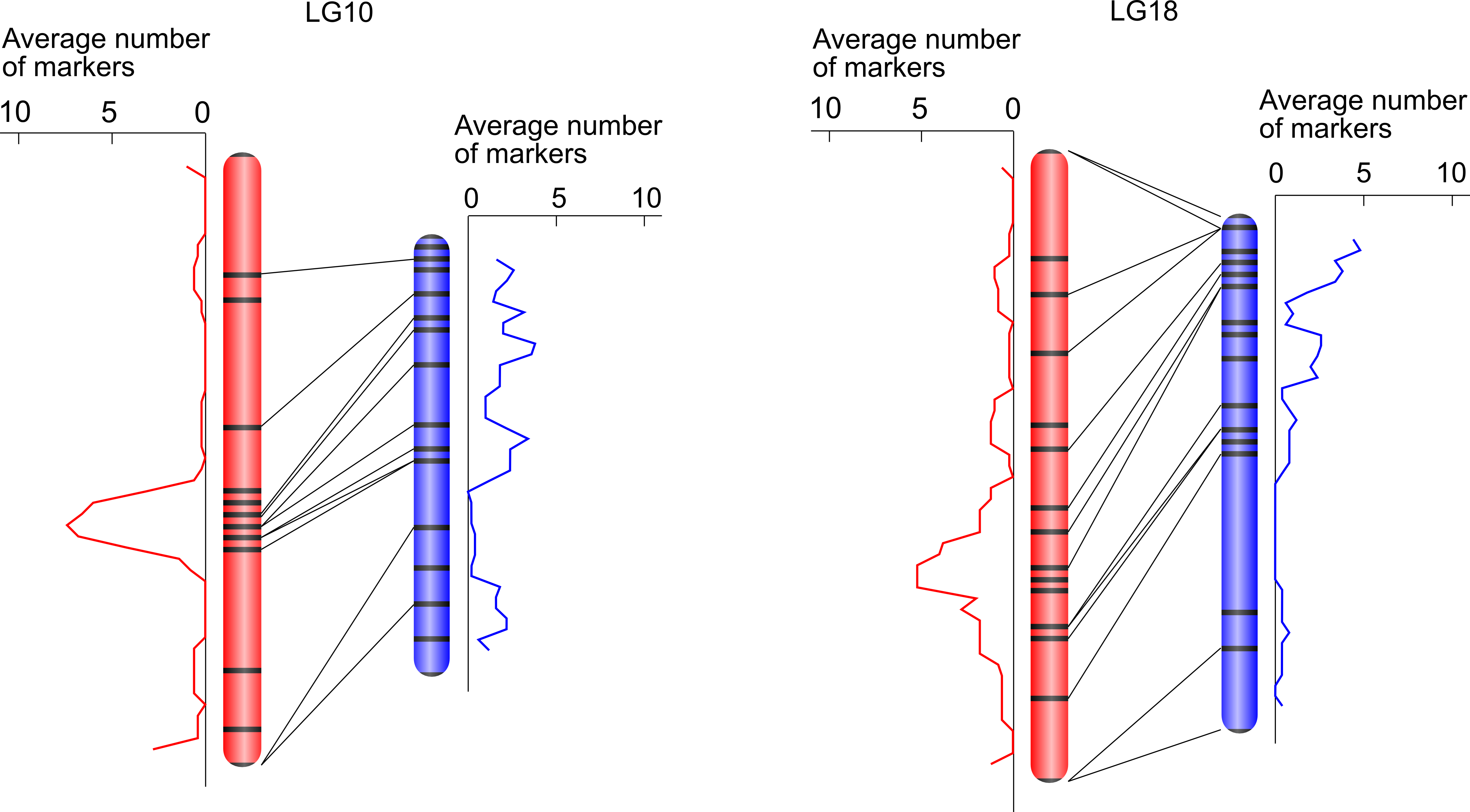


**Figure S7.** *-Continued.*


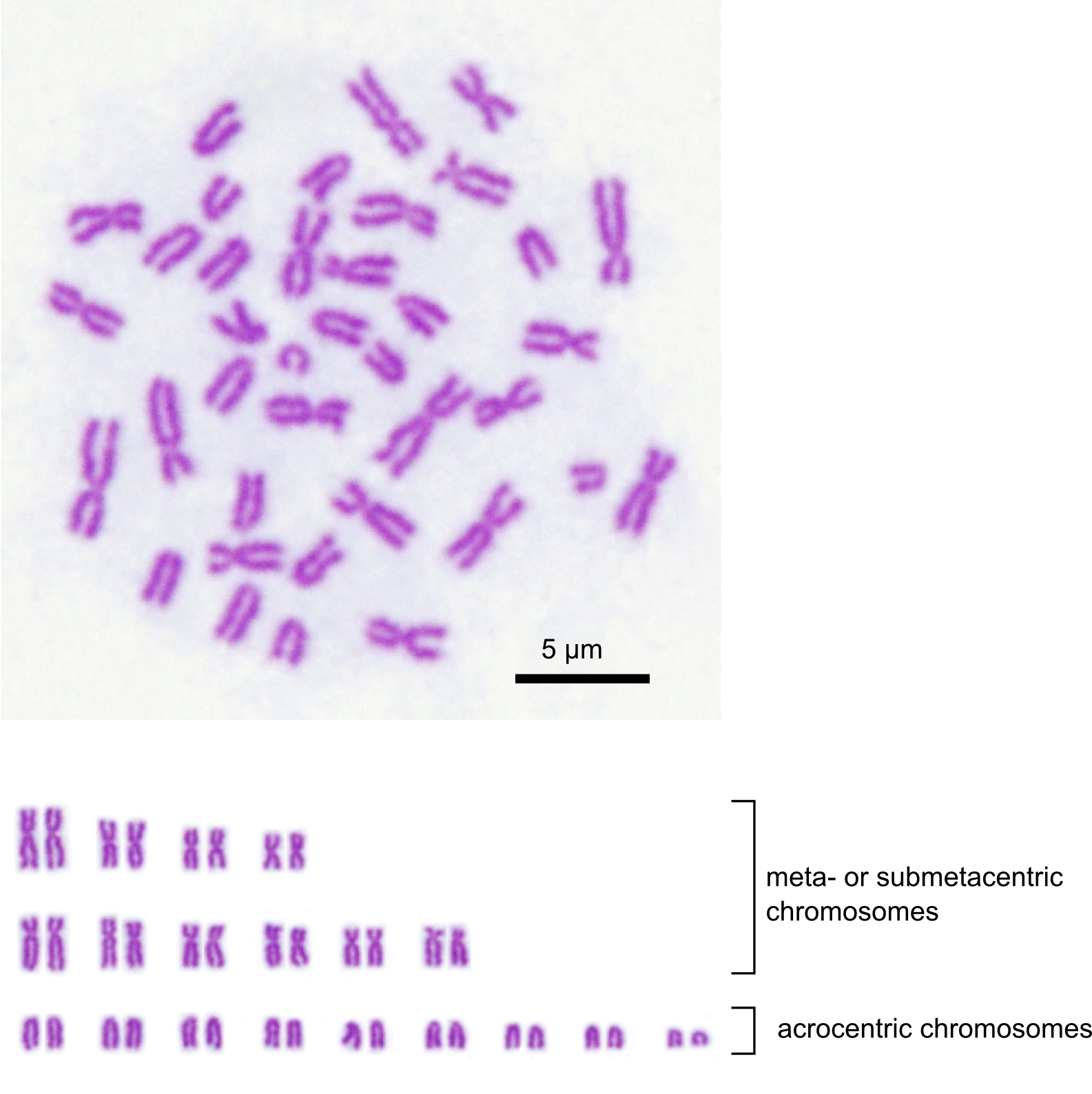


**Figure S8. Karyotype of the Japanese eel.** The chromosome preparation was obtained from peripheral blood leucocytes. Scale bar represents 5 µm. The karyotype of Japanese eel consists of 10 meta- or submetacentric and nine acrocentric chromosome pairs.
