# Supplementary material for: A ddRAD-based genetic map and its integration with the genome assembly of Japanese eel (Anguilla japonica) provides insights into genome evolution after the teleost-specific genome duplication
Source: BMC Genomics. 2014 Mar 26;15:233. doi: 10.1186/1471-2164-15-233 (PMC3986909; doi:10.1186/1471-2164-15-233)
Supplement: Additional file 4 — Comparison of ddRAD-seq and RAD-seq approaches. [file 1471-2164-15-233-S4.docx]

**Additional file 4. Comparison of ddRAD-seq and RAD-seq approaches.**

**Method of RAD library preparation**

A RAD library was constructed from genomic DNAs of the parents. Briefly, 500 ng of DNA template from the parents was digested with BamHI-HF (10 units per reaction, New England Biolabs Inc., Beverly, MA, USA) at 37°C for 1 h. Fragmented samples were purified using the MinElute PCR purification kit (Qiagen GmbH, Hilden, Germany). Purified samples were then ligated to CS1-tagged adapter (Fluidigm, South San Francisco, CA, USA). After ligation, each sample was purified using Agencourt AMPure XP beads (Beckman Coulter Inc.). The ligated DNA samples were sheared using a Covaris S220 Series (Covaris Pty Ltd, Bankstown Square, NSW 2200, Australia) into an average size of 200 bp. Sheared samples were then ligated to P1 adapter of Ion PGM (Life Technologies Corporation, Carlsbad, CA, USA). After second ligation, each sample was purified using Agencourt AMPure XP beads. Ligated samples were size-selected using the Pippin Prep (2% agarose cartridge, Sage Science, Beverly, MA, USA) under a “broad” setting with a mean of 250 bp and range of 220-280 bp. After size-selection, each sample was purified using Agencourt AMPure XP beads. Size-selected samples was purified using Agencourt AMPure XP beads and amplified using KAPA HiFi polymerase (KAPA biosystems, Woburn, MA, USA) with CS1-tagged TS forward primer (Fluidigm) and library amplification primer mix of Ion PGM (Life Technologies Corporation). The following PCR protocol was used: initial denaturation at 95°C for 5 min; 10 cycles of 95°C for 15 sec, 60°C for 30 sec and 72°C for 1 min; followed by a final extension period at 72°C for 3 min. The amplified libraries were purified using Agencourt AMPure XP beads and quantified using the Qubit fluorometer with a Quant-it dsDNA HS kit (Invitrogen, Carlsbad, CA, USA). The libraries of the parents were then pooled. The quality, size and concentration of the pooled libraries were finally determined using the 2100 Bioanalyzer with a high sensitivity DNA chip (Agilent technologies, Waldbronn, Germany). After library preparation, PGM sequencing, read processing, clustering and reference mapping were performed as described the methods of the main text.

**Calculation of the number of RAD loci and the region recovery**

To compare the ddRAD-seq and RAD-seq approaches, we calculated the number of RAD loci and the region recovery from random sampling of 1,500,000 tags from each parent of both approaches. For the ddRAD-seq, a total of 3,000,000 ddRAD-tags from the parents were clustered into 26,981 RAD loci (average cluster size was 111) (Additional file 2: Table S8). Of these, 10,614 loci were the cluster size of ≥50, 11,329 loci of ≥40, 12,190 loci of ≥30, 13,407 loci of ≥20, and 5,241 loci of singletons. All tags mapped to each dataset of the RAD loci under each filtration condition of the cluster size (Additional file 2: Table S8). Consequently, ≥85% of all tags were sufficient to recover >10,000 regions at over 100× under all filtration conditions. In contrast, for the RAD-seq, a total of 3,000,000 RAD-tags from the parents were clustered into 206,841 RAD loci (average cluster size was 14). Of these, 2,413 loci were the cluster size of ≥50, 3,413 loci of ≥40, 9,275 loci of ≥30, 48,090 loci of ≥20, and 29,937 loci of singletons. When the cluster size was ≥30, the number of RAD loci was similar to that of ddRAD-seq; however, the average depth was very low compared with the ddRAD-seq (Additional file 2: Table S8).
